# Protein Arginine Methyltransferase PRMT5 Regulates Fatty Acid Metabolism and Lipid Droplet Biogenesis in White Adipose Tissues

**DOI:** 10.1002/advs.202002602

**Published:** 2020-10-16

**Authors:** Zhihao Jia, Feng Yue, Xiyue Chen, Naagarajan Narayanan, Jiamin Qiu, Sabriya A. Syed, Anthony N. Imbalzano, Meng Deng, Peng Yu, Changdeng Hu, Shihuan Kuang

**Affiliations:** ^1^ Department of Animal Sciences Purdue University West Lafayette IN 47907 USA; ^2^ Department of Agricultural and Biological Engineering Purdue University West Lafayette IN 47907 USA; ^3^ Bindley Bioscience Center Purdue University West Lafayette IN 47907 USA; ^4^ Department of Biochemistry and Molecular Pharmacology University of Massachusetts Medical School Worcester MA 01605 USA; ^5^ West China Biomedical Big Data Center West China Hospital Sichuan University Chengdu 610041 China; ^6^ Medical Big Data Center Sichuan University Chengdu 610041 China; ^7^ Department of Medicinal Chemistry and Molecular Pharmacology Purdue University West Lafayette Indiana 47907 USA; ^8^ Purdue University Center for Cancer Research West Lafayette Indiana 47907 USA

**Keywords:** BSCL2, lipodystrophy, methylation, type 2 diabetes, adipocytes

## Abstract

The protein arginine methyltransferase 5 (PRMT5) is an emerging regulator of cancer and stem cells including adipogenic progenitors. Here, a new physiological role of PRMT5 in adipocytes and systemic metabolism is reported. Conditional knockout mice were generated to ablate the *Prmt5* gene specifically in adipocytes (Prmt5^AKO^). The Prmt5^AKO^ mice exhibit sex‐ and depot‐dependent progressive lipodystrophy that is more pronounced in females and in visceral (than subcutaneous) white fat. The lipodystrophy and associated energy imbalance, hyperlipidemia, hepatic steatosis, glucose intolerance, and insulin resistance are exacerbated by high‐fat‐diet. Mechanistically, Prmt5 methylates and releases the transcription elongation factor SPT5 from Berardinelli‐Seip congenital lipodystrophy 2 (*Bscl2*, encoding Seipin) promoter, and Prmt5^AKO^ disrupts Seipin‐mediated lipid droplet biogenesis. Prmt5 also methylates Sterol Regulatory Element‐Binding Transcription Factor 1a (SREBP1a) and promotes lipogenic gene expression, and Prmt5^AKO^ suppresses SREBP1a‐dependent fatty acid metabolic pathways in adipocytes. Thus, PRMT5 plays a critical role in regulating lipid metabolism and lipid droplet biogenesis in adipocytes.

## Graphical abstract

PRMT5 regulates protein function through methylating arginine residues. This study identifies two new functions of PRMT5 in adipocytes. First, PRMT5 methylates SPT5 to promote transcription of *Bscl2* gene, whose mutation causes Bernardinelli‐Seip Congenital Lipodystrophy in humans. Second, PRMT5 methylates SREBP1a to regulate fatty acid metabolism. Mice lacking PRMT5 in adipocytes develop progressive lipodystrophy and insulin resistance, resembling *BSCL2*‐mutation disease.

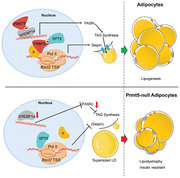

## 1 Introduction

Adipose tissue (AT) has the ability to store or oxidize excess lipids and is beneficial for the whole body energy homeostasis. Dysfunction of AT can result in insulin resistance and other metabolic complications in patients with too much or too little body fat, referred to as obesity and lipodystrophy, respectively.^[^ ^1^ ^]^ There are two types of AT: brown adipose tissue (BAT) and white adipose tissue (WAT). BAT dissipates glucose and fatty acids to generate heat, and thus it has an important role in cold‐ and diet‐induced thermogenesis.^[^ ^2^ ^]^ WAT is the main organ responsible for fat storage and serves as an indispensable endocrine organ that regulates systemic energy homeostasis. WAT is located in many distinct depots that arise from different progenitor cell populations, and WAT depots of different origin have distinct metabolic properties and adipokine secretion profiles.^[^ ^3^ ^]^ The subcutaneous WAT accounts for the majority of total AT in humans and has metabolic protective effects.^[^ ^4^ ^]^ The visceral WAT is, in contrast, associated with risks of metabolic diseases.^[^ ^5^ ^]^ White adipocytes are highly plastic and can uptake, esterificate and store excess lipids in the form of triacylglycerols (TAG) within lipid droplets (LDs), as well as undergo lipolysis to provide energy when nutrition is limited.^[^ ^6^ ^]^ Thus, understanding how lipid storage and utilization are regulated will provide a foundation for the development of therapeutics to overcome obesity and lipodystrophy caused by dysfunction of AT.

LDs are ubiquitous, highly dynamic cellular organelles responsible for storage and release of neutral lipids. Depending on metabolic demands, LDs undergo fascinating cycles of biogenesis and degradation.^[^ ^7^, ^8^ ^]^ Several human pathologies, such as Type 2 Diabetes (T2D) and non‐alcoholic fatty liver disease, are associated with the dysregulation of the life cycle and physiological functions of LDs.^[^ ^9^ ^]^ Thus, maintaining the homeostasis of LDs is critical for preventing ectopic lipid deposition and lipotoxicity. A favorite model for LD biogenesis includes several steps. First, neutral lipids (mostly TAG and sterol esters) are synthesized within the bilayer of endoplasmic reticulum (ER) membrane and accumulated to form an oil lens. Next, as TAG accumulates and the lipid lens grows, Seipin and other LD biogenesis factors are recruited to facilitate the budding of the lipid lens from ER into the cytosol, forming a nascent LD.^[^ ^8^, ^9^ ^]^ After initial budding, newly formed LDs retain a functional connectivity with the ER, and keep expanding through fusion or local lipid synthesis.^[^ ^10^ ^]^ Finally, small LDs free from the ER membrane can also coalesce to form large LDs. Seipin is a widely conserved oligomeric ER transmembrane protein that is required for correct LD budding.^[^ ^11^ ^]^ Seipin deficiency causes delayed LD formation and lower incorporation of proteins and lipids into the LDs, resulting in LDs that are very small and aggregated, and sometimes supersized.^[^ ^12^ ^]^ Although increasing evidence has shown how Seipin controls LD biogenesis and affects lipid metabolism, little is known about its transcriptional regulatory mechanism.

Arginine methylation catalyzed by PRMT family enzymes is a post‐translational modification in histone and non‐histone proteins. Arginine methylation is known to affect numerous cellular activities, including RNA processing, DNA repair, protein‐protein interactions and gene regulation.^[^ ^13^ ^]^ PRMT enzymes are classified depending on the type of methylation they catalyze: *ω*‐*N* ^G^‐monomethylarginine, asymmetric *ω*‐*N* ^G^ and *N* ^G^‐dimethylarginine, and symmetric *ω*‐*N* ^G^ and *N*′^G^‐dimethylarginine (sDMA). PRMT5 is a major type II arginine methyltransferase that catalyzes sDMA on multiple substrates.^[^ ^14^ ^]^ PRMT5 is brought to the forefront for its function in promoting tumor progression, and mounting evidence from mouse models demonstrates that PRMT5 inhibition can decrease the growth of many types of tumors including mantle cell lymphoma, acute myeloid lymphoma, chronic myeloid lymphoma, B‐cell lymphoma, glioma, and breast cancer.^[^ ^15^, ^16^ ^]^

Despite the well‐understood oncogenic role of PRMT5, its function outside the cancer field is just beginning to be elucidated. Recent studies have reported that PRMT5 regulates adipogenesis through controlling adipogenic and lipogenic gene expression in 3T3‐L1 cells,^[^ ^17^, ^18^ ^]^ but the in vivo physiological function of PRMT5 in adipose tissues is unknown. In the present study, by employing the conditional *Prmt5* knockout mouse model, we found that PRMT5 is required for white adipose tissue homeostasis and function. Deletion of *Prmt5* disrupts fatty acid metabolism and impairs lipid droplet biogenesis in white adipocytes, which causes age‐dependent fat loss and lipodystrophy, and eventually leads to insulin resistance and systemic metabolic syndrome. Mechanically, PRMT5 controls lipid droplet formation through methylation of SPT5, which transcriptionally regulates *Bscl2* expression. Meanwhile, PRMT5 promotes fatty acid biosynthesis through targeting SRBEP1a. Our study for the first time reports the physiological function of PRMT5 in AT and identifies a novel regulatory pathway controlling *Bscl2* gene expression and LD biogenesis, SREBP1a and lipid metabolism. These results provide insights into the role of PRMT5 in lipid metabolism, lipodystrophy and non‐obese T2D.

## 2 Results

### 2.1 Adipocyte‐Specific Knockout of *Prmt5* Leads to Age‐Dependent Losses in Fat Mass

**Figure 1:**
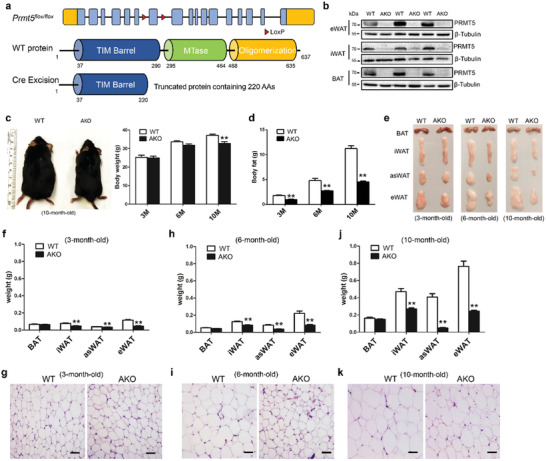
Adipocyte‐specific knockout of *Prmt5* (Prmt5^AKO^) leads to loss of fat mass in white adipose tissues. a) Targeting strategy for conditional knockout of Prmt5. Upper: *Prmt5* gene structure showing exons (blue boxes), LoxP insertions (red triangles) and 5′ and 3′ untranslated regions (orange boxes). Middle: PRMT5 protein domain structure with amino acids numbers labeled. MTase is the catalytic domain. Lower: Cre‐mediated excision of LoxP‐flanked exon results in reading frame shift and a premature translational stop codon, generating a truncated protein containing only partial of the TM barrel domain. b) Efficient reduction of PRMT5 protein levels in epididymal White Adipose Tissue (eWAT) and inguinal White Adipose Tissue (iWAT), and brown adipose tissue (BAT) of the 2 month old male Prmt5^AKO^ (AKO) mice. c) Representative images of male WT (Prmt5^flox/flox^) and Prmt5^AKO^ mice at 10 month old (left panel), showing progressive reduction in body weight of the Prmt5^AKO^ mice relative to WT mice (right panel). d) Prmt5^AKO^ leads to progressive loss of total body fat, relative to fat mass in WT mice. c,d) *n* = 8, 6, and 4 pairs of male mice for 3‐, 6‐, and 10‐month‐old, respectively. e) Representative images of BAT and WAT depots from male mice showing progressive mass reduction of *Prmt5* KO WAT with age. f,h,j) Weights of various BAT and WAT (iWAT, eWAT and anterior subcutaneous White Adipose Tissue, asWAT) depots from male mice at different ages, as shown in (e). g,i,k) H&E staining of eWAT sections from WT (Prmt5^flox/flox^) and Prmt5^AKO^ male mice at 3, 6, and 10month old. Scale bar: 50 µm. Data represent mean ± s.e.m. (*t*‐test: **p < 0.01).

To investigate the role of PRMT5 in adipocytes, we generated adipocyte‐specific *Prmt5* knockout mice by crossing the *Prmt5^f/f^* mice with *Adipoq‐Cre* mice (**Figure** **1**a). In the Prmt5^AKO^ mice, the exon 7 of *Prmt5* is deleted, leading to premature translational stop and generation of a truncated peptide lacking the key functional domains including the methyltransferase (MTase) domain (Figure 1a). In this model, *Prmt5* should be deleted exclusively in adipocytes as *Adipoq‐Cre* specifically marks all mature adipocytes.^[^ ^19^ ^]^ We confirmed the efficient and specific reduction of PRMT5 protein levels in both BAT and WAT by Western blotting (Figure 1b), but not in muscle tissues (Figure S1a, Supporting Information). We also confirmed that mRNA levels of *Prmt5* were specifically reduced in mature adipocytes but not in SVF preadipocytes that do not express *Adipoq* (Figure S1b, Supporting Information).

The Prmt5^AKO^ mice were born at the expected Mendalian ratio, and were morphologically indistinguishable from their WT littermates. However, as the Prmt5^AKO^ mice grew, they began to appear leaner in an age‐dependent manner (Figure 1c). At 10 month old, the body weights of Prmt5^AKO^ mice were significantly lighter than that of WT mice (Figure 1c). EchoMRI body composition scanning showed progressively less accumulation of total body fat mass in Prmt5^AKO^ mice compared with WT control (Figure 1d). At 3‐, 6‐, and 10 month old, the fat masses of the Prmt5^AKO^ mice were 52%, 57%, and 60% less than those of WT mice, respectively (Figure 1d). Inspection of various fat depots revealed that fat mass difference was specific to WAT but not BAT (Figure 1e), and more prominent in visceral eWAT and anterior subcutaneous asWAT than in inguinal iWAT (Figure 1f,h,j). Further inspection demonstrated that reduced gain of fat mass in Prmt5^AKO^ mice was detectable as early as 8 week old (Figure S2a–c, Supporting Information). Interestingly, the reduction in fat mass gain appeared to be more prominent in female than in male Prmt5^AKO^ mice (Figure S2c, Supporting Information). The size of eWAT adipocytes was also obviously smaller in 6 and 10 month old Prmt5^AKO^ mice than age‐matched WT mice (Figure 1g,i,k). Taken together, deletion of Prmt5 in adipocytes leads to progressive age‐, sex‐, and depot‐dependent reductions in WAT mass accumulation.

### 2.2 Prmt5^AKO^ Mice Are Protected from High‐Fat Diet‐Induced Obesity but Insulin Resistant

**Figure 2:**
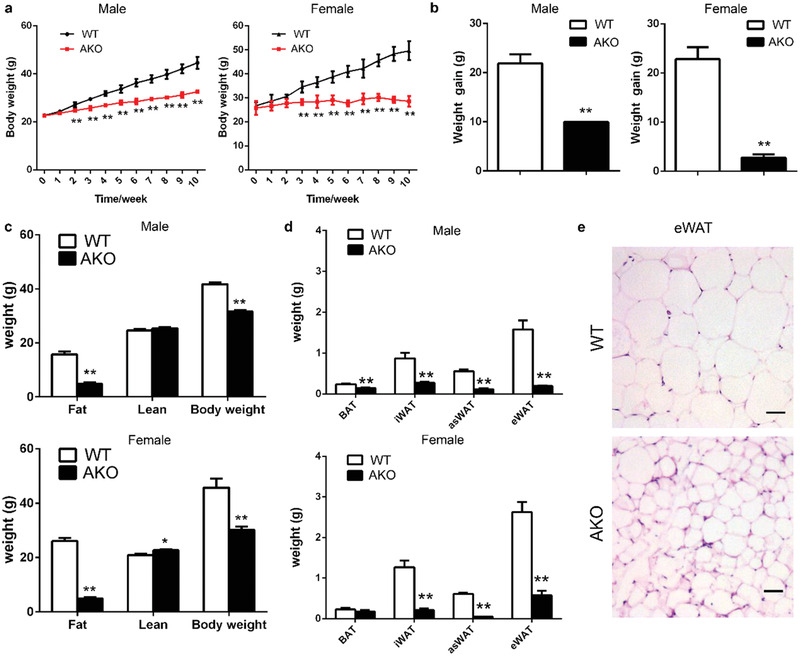
Prmt5^AKO^ mice are protected from high‐fat diet (HFD)‐induced fat accumulation. a) Growth curve of male (left) and female (right) WT (Prmt5^flox/flox^) and Prmt5^AKO^ mice during 10 week of HFD feeding. b) Weight gain of male (left) and female (right) WT (Prmt5^flox/flox^) and Prmt5^AKO^ mice after 10 week of HFD feeding. a,b) *n* = 8 and 9 pairs of mice for male and female mice, respectively. c) Body weight, fat mass, and muscle mass of male (upper) and female (lower) WT (Prmt5^flox/flox^) and Prmt5^AKO^ mice after 12 week of HFD feeding. *n* = 8 and 4 pairs of mice for male and female mice, respectively. d) WAT and BAT absolute weights from male (upper panel) and female (lower panel) WT (Prmt5^flox/flox^) and Prmt5^AKO^ mice after 12 week of HFD, *n* = 5 pairs of mice. e) H&E staining of eWAT sections from male WT (Prmt5^flox/flox^) and Prmt5^AKO^ mice after 12 week of HFD. Scale bar: 50 µm. Data represent mean ± s.e.m. (*t*‐test: * *p* < 0.05, **p < 0.01).

In view of their reduced accumulation of WAT, we predicted that Prmt5^AKO^ mice should be more resistant to high‐fat diet (HFD)‐induced obesity than the WT mice. Indeed, both male and female Prmt5^AKO^ mice were consistently leaner and gained less body weight after being fed with HFD for 2 weeks (**Figure** **2**a,b) even though their food intake was similar to that of their WT littermates (data not shown). The resistance to body weight gain was also more obvious in female than male Prmt5^AKO^ mice (Figure 2a,b). After fed with HFD for 12 weeks, the male and female Prmt5^AKO^ mice gained only 46% and 12% of the body weight gained by the WT male and female mice, respectively (Figure 2b). EchoMRI body composition scanning showed that the differences in body weights between Prmt5^AKO^ and WT mice were mainly due to differences in fat mass (Figure 2c), Specifically, the male and female Prmt5^AKO^ mice gained 10.9 ± 1.9 g and 21.2 ± 1.8 g less fat compared to their sex‐matched WT littermates (Figure 2c). We also isolated various fat depots (Figure S3a, Supporting Information) and measured their weights. All Prmt5^AKO^ WAT depots were significantly lighter than corresponding depots in sex‐matched WT mice, and the differences were more prominent in asWAT and eWAT (Figure 2d and Figure S3b, Supporting Information). The results also revealed strikingly more resistance to HFD‐induced obesity by the Prmt5^AKO^ female than male mice. The overall size of eWAT adipocytes was also obviously smaller in Prmt5^AKO^ mice than WT mice after HFD, and the size of adipocytes was very heterogeneous in the Prmt5^AKO^ mice (Figure 2e). The mass of BAT was not changed in the Prmt5^AKO^ mice (Figure S3b, Supporting Information), nor were the lipid droplet size and the ultrastructure of brown adipocytes altered (Figure S4a–c, Supporting Information). These data demonstrate that Prmt5^AKO^ mice exhibit sex‐ and depot‐ dependent resistance to HFD‐induced fat accumulation.

**Figure 3:**
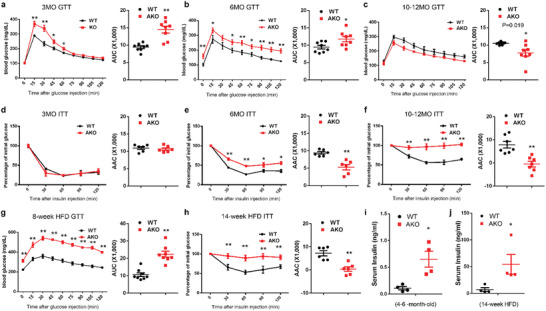
Impaired glucose tolerance and insulin sensitivity in Prmt5^AKO^ mice. a–c) Blood glucose concentrations during glucose tolerance tests (GTT) performed on a) 3, b) 6, and c) 10–12 months old male WT (Prmt5^flox/flox^) and Prmt5^AKO^ mice (left). Area under curve (AUC) calculated based on data in left panel (right). *n* = 8, 8, and 7 pairs of mice for 3, 6, and 10–12 month old, respectively. d–f) Percentage changes of blood glucose concentrations during insulin tolerance tests (ITT) performed on d) 3‐, e) 6‐, and f) 10–12 month old male WT (Prmt5^flox/flox^) and Prmt5^AKO^ mice (left). Area above curve (AAC) calculated based on data in left panel (right). *n* = 6, 6, and 7 pairs of mice for 3, 6, and 10–12 month old, respectively. g) Blood glucose concentrations during glucose tolerance tests (GTT) performed on male WT (Prmt5^flox/flox^) and Prmt5^AKO^ mice after 8 week of HFD (left). AUC calculated based on data in left panel (right), *n* = 8 pairs of male mice. h) Percentage changes of blood glucose concentrations during insulin tolerance tests (ITT) performed on male WT (Prmt5^flox/flox^) and Prmt5^AKO^ mice after 14 week of HFD (left). AAC calculated based on data in left panel (right), *n* = 6 pairs of male mice. i,j) Blood insulin level of 4–6 months old male mice i)before and j) after 14 week HFD feeding, *n* = 4 pairs of male mice. Data represent mean ± s.e.m. (*t*‐test: *p < 0.05, **p < 0.01).

We also examined how reduction of WAT in the Prmt5^AKO^ mice affects their glucose tolerance and insulin sensitivity. We first conducted glucose tolerance tests (GTT) to determine glucose disposal after intraperitoneal (i.p.) injection of glucose into Prmt5^AKO^ and WT mice at various ages (**Figure** **3**a–c). At 3‐ and 6‐month old, the Prmt5^AKO^ mice had higher blood glucose levels than the WT littermates after injection of glucose (Figure 3a,b). Higher fasting glucose levels were also observed in 6 month old Prmt5^AKO^ mice compared with WT mice (Figure 3b). Area under curve (AUC) of the Prmt5^AKO^ mice were also significantly larger than those of WT mice at corresponding age groups (Figure 3a,b), suggesting that young Prmt5^AKO^ mice are glucose intolerant. Interestingly, however, the Prmt5^AKO^ mice no longer exhibit glucose intolerance at 10–12 months old, and had lower levels of blood glucose and smaller AUC compared to WT mice (Figure 3c). In addition, 10‐month‐old Prmt5^AKO^ mice had a larger muscle mass compared with WT mice (Figure S5a, Supporting Information). We next performed insulin tolerance tests (ITT) to determine how blood glucose level changes in response to insulin injection (Figure 3d–f). We observed a different response than the GTT response. Specifically, young Prmt5^AKO^ mice (3‐month‐old) exhibited normal insulin sensitivity, as manifested by similar rates of insulin‐mediated glucose clearance and identical area above the curve (AAC) in Prmt5^AKO^ and WT (Figure 3d). At 6 month old, however, Prmt5^AKO^ mice showed slower glucose clearance than WT mice, which was also confirmed by a significantly smaller AAC (Figure 3e). The 10–12 months old Prmt5^AKO^ mice were even more insulin resistant as shown by the complete lack of changes in blood glucose levels in response to insulin injection (Figure 3f). Therefore, the Prmt5^AKO^ mice exhibit age‐dependent deterioration of insulin sensitivity. We further measured GTT and ITT after HFD feeding, which exacerbated the glucose tolerance and insulin sensitivity in both Prmt5^AKO^ and WT mice (Figure 3g,h). Nevertheless, Prmt5^AKO^ mice displayed worse glucose tolerance and insulin sensitivity than WT mice after HFD treatment (Figure 3g,h and Figure S5b,c, Supporting Information). In addition, the serum insulin level of 4–6 months old Prmt5^AKO^ mice was about 7‐fold higher than that of WT littermates (Figure 3i). Strikingly, after 14 weeks of HFD treatment, the serum insulin level of male Prmt5^AKO^ mice reached 54.5 ± 18.3 ng mL^−1^, which was almost 8‐fold elevation compared to the 7.3 ± 3.4 ng mL^−1^ level in the WT mice (Figure 3j). These data together demonstrate that despite having a lean body mass, the Prmt5^AKO^ mice progressively develop insulin resistance that is exacerbated by HFD feeding.

The lean body mass and insulin resistance phenotype of the Prmt5^AKO^ mice resemble the clinical manifestation of human non‐obese T2D patients. This prompted us to examine if *PRMT5* level is correlated with non‐obese T2D. We retrieved data from published studies,^[^ ^20^, ^21^ ^]^ in which *PRMT5* expression data in WAT from lean and obese patients with different glucose tolerance, as well as from obese and non‐obese T2D patients are available. The results showed that *PRMT5* expression was not significantly different between healthy lean and obese humans (Figure S6a, Supporting Information), but exhibited a trend to decrease during the development of glucose intolerance (Figure S6b, Supporting Information). Notably, *PRMT5* expression levels are significantly lower in non‐obese T2D than obese T2D patients (Figure S6c, Supporting Information). These results establish a negative correlation between *PRMT5* expression level and non‐obese T2D in humans, as we shown in mice.

### 2.3 Prmt5^AKO^ Mice and Adipocytes Exhibit Lower Metabolic Rates

**Figure 4:**
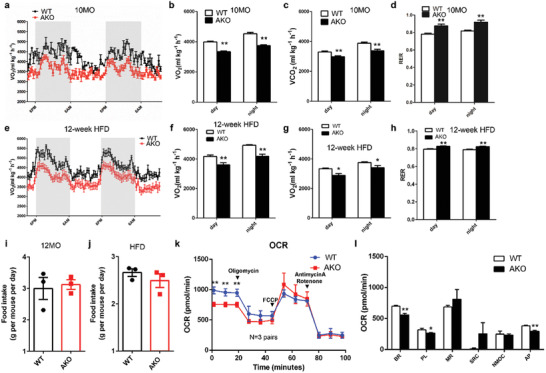
Prmt5^AKO^ mice exhibit lower metabolic rate and lower basal respiration in Prmt5 deficient adipocytes. a) O2 consumption measured by an indirect calorimetry and normalized to lean mass is shown for a 48 h cycle of 10‐month‐old male WT (Prmt5^flox/flox^) and Prmt5^AKO^ mice. b–d) Average day and night O2 consumption (b) VO2, CO2 production c) VCO2 and d) RER. a–d) *n* = 5 pairs of 10‐month‐old male mice. e) O2 consumption measured same as above on male WT (Prmt5^flox/flox^) and Prmt5^AKO^ mice after 12 week of HFD. f–h) Average day and night O2 consumption f) VO2, CO2 production g) VCO2 and h) RER. e–h) *n* = 6 pairs of male mice after 12 week of HFD. i,j) Food intake of WT (Prmt5^flox/flox^) and Prmt5^AKO^ mice during i) control‐diet or j) HFD feeding. *n* = 3 groups of male mice for control‐diet (12‐month‐old) and HFD feeding, respectively. Each dot represents the average food consumption per day of two mice. k,l) Seahorse‐based measurement of oxygen consumption rate (OCR) on white adipocytes differentiated from preadipocytes isolated from iWAT of 8 week old WT (Prmt5^flox/flox^) and Prmt5^AKO^ mice. k) OCR was measured at basal state and after sequential addition of Oligomycin, FCCP, and Antimycin A/Rotenone to determine the l) Basal Respiration (BR), Maximal Respiration (MR), Spare Respiratory Capacity (SRC), Non‐Mitochondrial Oxygen Consumption (NMOC), and ATP Production (AP), respectively, cells were isolate from the iWAT of *n* = 4 pairs of male WT (Prmt5^flox/flox^) and Prmt5^AKO^ mice, measured in five technical replicates. Data represent mean ± s.e.m. (*t*‐test: *p < 0.05, **p < 0.01).

We next examined the metabolic rate of Prmt5^AKO^ mice using an indirect calorimetry approach. Prmt5^AKO^ mice had lower levels of oxygen (O_2_) consumption and carbon dioxide (CO_2_) production than WT mice at 10‐month‐old (**Figure** **4**a–c and Figure S7a, Supporting Information). The respiratory exchange ratio (RER) was increased in Prmt5^AKO^ mice (Figure 4d), suggesting increased utilization of carbohydrates over fatty acids. Prmt5^AKO^ mice also expended less energy than WT mice, but not at a significant level (Figure S7b, Supporting Information). After fed with HFD for 12 weeks, male Prmt5^AKO^ mice also had lower rates of O_2_ consumption and CO_2_ production than WT mice (Figure 4e–g and Figure S7c, Supporting Information). RER was also higher in Prmt5^AKO^ mice than in WT mice after HFD (Figure 4h). The heat production was also significantly lower in male Prmt5^AKO^ mice than WT mice after HFD treatment (Figure S7d, Supporting Information). Similar results were observed in female Prmt5^AKO^ mice after HFD treatment (Figure S7e–h, Supporting Information). The differences in energy expenditure between WT and Prmt5^AKO^ mice were not due to energy intake, as the WT and Prmt5^AKO^ mice consumed comparable amounts of food (Figure 4i,j).

To examine whether the systemic metabolic changes were due to defective energy expenditure of adipocytes, we analyzed oxygen consumption rate (OCR) of adipocytes differentiated from Prmt5^AKO^ and WT SVF preadipocytes. The OCR associated with basal respiration, proton leak and ATP production were all significantly decreased in Prmt5^AKO^ than in WT adipocytes (Figure 4k,l). These results together demonstrate that reduced metabolic rate of *Prmt5*‐null adipocytes contributes to systemic metabolic defects in Prmt5^AKO^ mice.

### 2.4 Defective Seipin‐Dependent Lipid Droplet Formation in *Prmt5* Knockout Adipocytes

**Figure 5:**
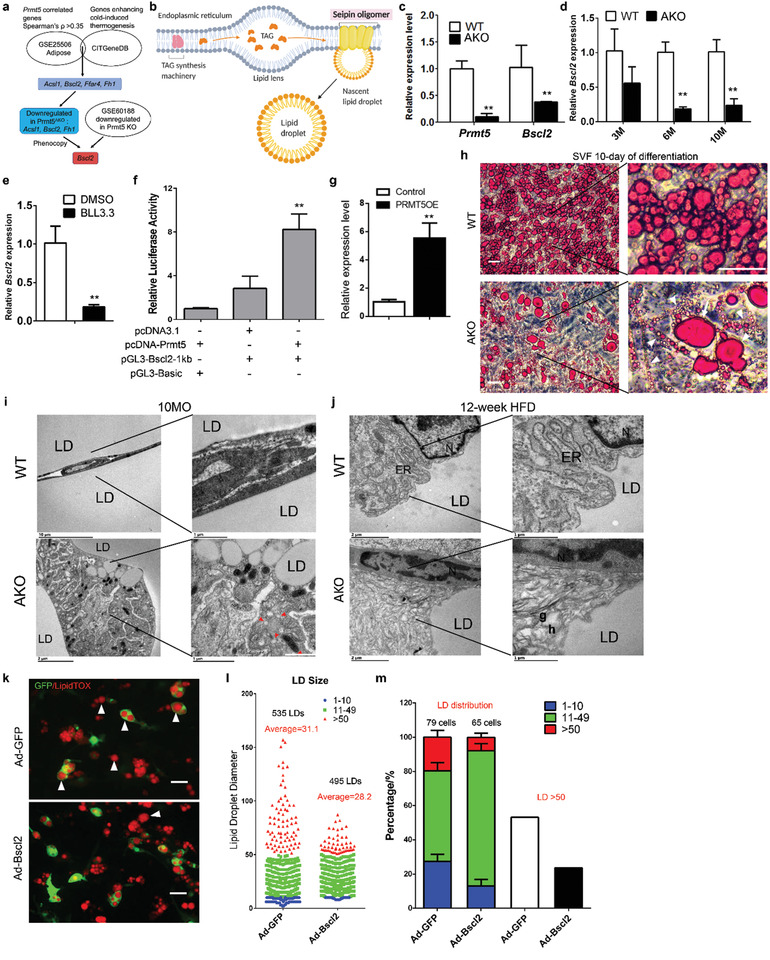
Prmt5^AKO^ leads to abnormal lipid droplet biogenesis due to downregulating *Bscl2* (Bscl2) expression. a) Prediction of *Bscl2* as a PRMT5 target gene based on gene expression and phenotypic correlations. b) Role of seipin in lipid droplet biosynthesis at the endoplasmic reticulum (ER). c) *Prmt5* and *Bscl2* expression level in SVF preadipocytes from iWAT of WT (Prmt5^flox/flox^) and Prmt5^AKO^ mice after 10 days of differentiation in vitro, *n* = 3 pairs of male mice at 8 week old. d) Expression levels of *Bscl2* in the eWAT from 3‐, 6‐, and 10‐month‐old WT (Prmt5^flox/flox^) and Prmt5^AKO^ mice, *n* = 3 pairs of male mice. e) Pharmacological inhibition of PRMT5 (BLL3.3, 20 µm) reduces *Bscl2* expression in 3T3‐L1 cells. DMSO is vehicle control, *n* = 3. f) Luciferase assay of HEK‐293A cells co‐transfected with *Prmt5* and pGL‐Bscl2 1kb promoter (pGL3‐Bscl2‐1kb), *n*  =  4 biological replicates for each independent experiment. g) *Bscl2* mRNA levels in control and *Prmt5*‐overexpressed 3T3‐L1 cell lines, *n* = 3 biological repeats, and each sample was detected in three technical repeats. h) Oil red staining of SVF preadipocytes from male WT (Prmt5^flox/flox^) and Prmt5^AKO^ mice after 10 days of differentiation in vitro (4 days of induction medium followed by 6 days of differentiation medium). Arrows and arrowheads indicate abnormally large and small lipid droplets, respectively. Scale bar: 50 µm. i,j) Representative Transmission Electron Microscopy (TEM) images of eWAT from i) 10‐month‐old and j) 12‐week HFD fed WT (Prmt5^flox/flox^) and Prmt5^AKO^ mice, *n* = 3 pairs of male mice. k) Representative images of SVF preadipocytes from iWAT of male Prmt5^AKO^ mice after 10 day of differentiation treated with GFP‐expressing adenovirus (control) or Bscl2/GFP co‐expressing adenovirus, white arrowheads indicate the supersized lipid droplets, scale bar: 50 µm. l) Size distribution lipid droplets in GFP adenovirus or Bscl2/GFP adenovirus treated SVF preadipocytes from Prmt5^AKO^ mice after 10 day of differentiation. In total, 535 LDs and 495 LDs from six different Prmt5^AKO^ mice were counted. Five random microscopic images were captured and counted from either Ad‐GFP or Ad‐Bscl2/GFP groups. m) Average lipid droplet composition of different diameters in one cell and percentage of cells containing lipid droplet with diameter of >50 pixels from GFP adenovirus or Bscl2‐GFP adenovirus treated SVF preadipocytes from Prmt5^AKO^ mice after 10 days of differentiation. Data represent mean ± s.e.m. (*t*‐test: *p < 0.05, **p < 0.01). Diameters in l&m are in pixels, 1 pixel = 0.3223 µm.

To further understand how PRMT5 regulates lipid metabolism, we used a prediction method based on expression and phenotypic correlations (**Figure** **5**a). We first identified a small set of four common genes whose expression is positively correlated to *Prmt5* in AT based on the microarray dataset GSE25506^[^ ^22^ ^]^ and who are also candidate genes in our cold‐induced thermogenesis (CIT, a process involving lipid droplet dynamics) database CITGeneDB.^[^ ^23^ ^]^ We then validated the expression of the four genes and found that expression of *Acsl1*, *Bscl2*, and *Fh1* was downregulated in Prmt5^AKO^ AT. By analyzing RNA‐Seq dataset GSE60188,^[^ ^16^ ^]^ we also found that *Bscl2* is down‐regulated when *Prmt5* is perturbed.^[^ ^24^ ^]^ In addition, our Prmt5^AKO^ mice phenocopied the *Bscl2* knockout mice in lipodystrophy.^[^ ^25^ ^]^ The *Bscl2* gene encodes Seipin, a critical regulator of LD biogenesis following TAGs biosynthesis in the space between inner and outer membranes of ER (Figure 5b). Thus, we prioritized Seipin as a candidate downstream target of PRTM5.

To confirm if PRMT5 regulates Seipin, we examined *Bscl2* expression in response to *Prmt5* knockout. We isolated SVF cells from WT and Prmt5^AKO^ mice and differentiated them into adipocytes. The mRNA level of *Prmt5* was reduced by more than 80% in the Prmt5^AKO^ cells, and mRNA level of *Bscl2* was reduced by 60% (Figure 5c). *Bscl2* expression in eWAT of 6‐ and 10‐month‐old Prmt5^AKO^ mice was also significantly lower than that of age‐matched WT mice (Figure 5d). Moreover, pharmacological inhibition of PRMT5 using small molecule inhibitor BLL3.3 also reduced the expression of *Bscl2* in 3T3‐L1 adipogenic cells (Figure 5e). To examine the effect of PRMT5 on *Bscl2* expression directly, we subcloned the 1kb promoter region of *Bscl2* into the pGL3 plasmid (pGL3‐Bscl2‐1kb) and performed dual luciferase assay with or without *Prmt5* overexpression. The relative luciferase activity of cells co‐transfected with *Prmt5* was significantly higher than cells without *Prmt5* co‐transfection (Figure 5f). In addition, mRNA level of *Bscl2* was significantly increased in *Prmt5*‐overexpressing 3T3‐L1 cells (Figure 5g). These data demonstrate that PRMT5 positively regulates *Bscl2* expression.

We then examined the role of PRMT5 in lipid droplet biogenesis. We cultured SVF preadipocytes from subcutaneous WAT of Prmt5^AKO^ and WT mice, and induced the SVF cells to differentiate (IM 4d + DM 6d) into LD‐laden adipocytes. Strikingly, Prmt5^AKO^ adipocytes contained LDs that were highly heterogeneous in size (Figure 5h). Compared to the relative homogenous size of LDs in WT cells, many of the LDs in Prmt5^AKO^ adipocytes were super large or very small in size (Figure 5h, insets). Consistently, adipocytes differentiated from eWAT SVF cells of Prmt5^AKO^ mice also contained super large and very small LDs (Figure S8a,b, Supporting Information). We further examined the ultrastructure of LDs in eWAT adipocytes by transmission electron microscope (TEM). TEM confirmed the abnormal presence of numerous small LDs in the Prmt5^AKO^ adipocytes of 10‐month‐old mice, in contrast to unilocular large LDs normally seen in WT adipocytes (Figure 5i). Additional defective wrinkled LD membrane was observed in Prmt5^AKO^ adipocytes of 6‐ and 10‐month‐old mice, which is in contrast to the smooth LD membrane in WT mice (Figure S8c,d, Supporting Information). Notably, we also observed dramatic changes in ER structure of Prmt5^AKO^ mice after fed with HFD for 12 weeks (Figure 5j). In adipocytes of WT mice, the membrane structure and lumen of ER, and associated ribosomes and emerging LDs were clearly visible (Figure 5j). However, the ER membrane and lumen appeared highly disorganized in the Prmt5^AKO^ adipocytes (Figure 5j). The defective wrinkled LD membrane was also observed in Prmt5^AKO^ adipocytes after fed with HFD for 12 weeks (Figure S8e, Supporting Information). These observations point to a critical role of PRMT5 in LD biogenesis.

Seipin is an ER‐membrane protein key for LD biogenesis,^[^ ^9^ ^]^ we therefore asked if the defective LD biogenesis in Prmt5^AKO^ adipocytes is due to the reduced level of Seipin. To address the question, we performed Seipin gain‐of‐function studies in SVF cells isolated from the subcutaneous WAT from WT and Prmt5^AKO^ mice. We used adenovirus to express *GFP* (control) or *Bscl2/GFP* in the SVF cells, and Seipin protein levels were substantially elevated by the Bscl2‐adenovirus compared to GFP‐adenovirus infection (Figure S8f, Supporting Information). We then differentiated the SVF cells and used LipidTOX staining to visualize LDs. In WT SVF cells, *Bscl2* overexpression (marked by GFP) decreased the numbers of the extra‐large and very small LDs, but slightly increased the average size of LDs (Figure S8g,h, Supporting Information). In Prmt5^AKO^ SVF cells, *GFP* expression had no effects on LD composition, as abnormally large LDs were detected in both GFP^+^ and GFP^−^ cells (Figure 5k). Strikingly, *Bscl2* overexpression (marked by GFP) effectively reduced the abundance of cells containing abnormally large LDs, which were mostly found in non‐infected (GFP^−^) cells (Figure 5k). To quantify LD distribution, we classified LDs into large (>50 pixels), medium (10–50 pixels) and small (<10 pixels) groups. The quantitative analysis showed that *Bscl2* overexpression robustly reduced the size range by decreasing the size of abnormally large and increasing the size of very small LDs (Figure 5l). The average LD size was slightly decreased by *Bscl2* overexpression (Figure 5l). Consequently, the percentage of large and small LDs were both decreased while the percentage of medium size LDs was increased, by *Bscl2* overexpression, resulting in less cells with large LDs (Figure 5m). Therefore, *Bscl2* overexpression efficiently normalizes the size of LDs in Prmt5^AKO^ adipocytes. These data collectively provide compelling evidence that PRMT5 regulates LD biogenesis through regulating the level of *Bscl2*.

### 2.5 PRMT5 Promotes *Bscl2* Expression by Methylating SPT5 and Disassociating It from *Bscl2* TSS

**Figure 6:**
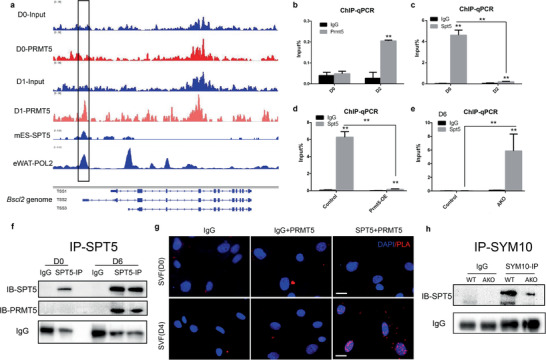
PRMT5 interacts with and methylates the transcriptional pausing factor SPT5 to affect its binding to the *Bscl2* TSS. a) ChIP‐sequencing results showing co‐occupancy of PRMT5, SPT5, and RNA polymerase II (POL2) at the same TSS region (boxed region) of *Bscl2* genome (shown in the bottom panel). Note that PRMT5 only occupies the region in differentiated (D1) but not undifferentiated (D0) 3T3‐L1 cell. SPT5 ChIP‐seq is based on results from mouse embryonic stem cell (mES) and POL2 ChIP‐seq is based on results from eWAT.^[^ ^27^, ^28^ ^]^ b,c) ChIP‐qPCR analysis of b) PRMT5 and c) SPT5 binding to the boxed region shown in a in undifferentiated (D0) and differentiated 3T3‐L1 cells (D2). d) SPT5 ChIP‐qPCR results on *Prmt5* overexpressing 3T3‐L1 cells and control cells. e) SPT5 ChIP‐qPCR of *Bscl2* promoter in SVF cells from WT (Prmt5^flox/flox^) and Prmt5^AKO^ mice after 6 day of differentiation in vitro. f) Lysates from undifferentiated (D0) and differentiated 3T3‐L1 cells (D6) were immunoprecipitated (IP) with SPT5 antibody and blotted with SPT5 and PRMT5 antibodies showing association with PRMT5 only in differentiated cells. g) Proximity ligation assay (PLA) using PRMT5 and SPT5 antibodies in WT undifferentiated (D0) and differentiated SVF preadipocytes (D4) showing PRMT5 interacts with SPT5 in nucleus only in differentiated cells. h) Lysates from eWAT of WT (Prmt5^flox/flox^) and Prmt5^AKO^ mice were immunoprecipitated (IP) with SYM10 antibody and blotted with SPT5 antibody. (*t*‐test: **p < 0.01).

To understand how PRMT5 regulates the expression of Seipin (*Bscl2)*, we overlapped known PRMT5 substracts from previous publications^[^ ^26^ ^]^ and regulators of *BSCL2* based on information in GeneCards (https://www.genecards.org), and identified SPT5 as a candidate target of PRMT5. Analysis of previously published chromatin immunoprecipitation (ChIP) data^[^ ^27^ ^]^ revealed that the transcription start site (TSS) region of *Bscl2* is occupied by SPT5 in mouse embryonic stem cell (mES) (**Figure** **6**a). Then we performed ChIP‐seq using a PRMT5 antibody to pull down genomic DNA from undifferentiated and differentiated 3T3‐L1 cells. A specific PRMT5 binding peak was identified at the same *Bscl2* TSS region as SPT5, but only in the differentiating cells (Figure 6a). The lack of PRMT5 binding in non‐differentiated cells may be due to the low PRMT5 expression in preadipocytes.^[^ ^17^ ^]^ Further analysis of previously published ChIP data^[^ ^28^ ^]^ identified a consensus binding peak of RNA polymerase II (POL2) at the same TSS region in mouse eWAT (Figure 6a). The SPT5 binding to this region appears to be evolutionarily conserved, as SPT5 ChIP‐seq data on human K562 cell line and ATAC‐seq of human subcutaneous adipose tissue^[^ ^29^ ^]^ also showed similar binding peaks at *BSCL2* TSS (Figure S9a, Supporting Information). These data reveal association of PRMT5, POL2, and SPT5 at the TSS of *Bscl2* gene.

We then performed ChIP‐qPCR assay using primers flanking the *Bscl2* TSS to validate the ChIP‐seq data. Consistently, *Bscl2* TSS was highly enriched by PRMT5 antibody after 2 days of differentiation, but not in undifferentiated 3T3‐L1 cells (Figure 6b). On the contrary, *Bscl2* TSS was highly enriched by SPT5 antibody in undifferentiated 3T3‐L1 cells, and the enrichment was reduced by 20‐fold after 2 days of differentiation (Figure 6c). We then established a stable 3T3‐L1 cell line overexpressing *Prmt5* (Figure S9b, Supporting Information), which showed a concomitant reduction of TSS enrichment by SPT5 antibody (Figure 6d). As binding specificity control, primers flanking the *MG53* (Chromosome 7) promoter region showed almost no enrichment by SPT5 antibody (Figure S9c, Supporting Information). We also isolated SVF preadipocytes from Prmt5^AKO^ and WT mice. There were no differences between WT and KO cells in *Seipin* TSS enrichment at D0 by PRMT5 (which has low expression in preadipocytes) or SPT5 antibody (Figure S9d,e, Supporting Information). At D6 (after differentiation), the *Bscl2* TSS enrichment by SPT5 antibody was significantly higher in Prmt5^AKO^ compared with WT cells (Figure 6e). Consistently, the *Bscl2* TSS enrichment by PRMT5 antibody was reduced by 5‐fold in Prmt5^AKO^ adipocytes compared with WT cells after 6 days of differentiation (Figure S9f, Supporting Information). Collectively, these results provide strong evidence that PRMT5 and SPT5 have mutually exclusive binding patterns on *Bscl2* TSS, and upregulation of PRMT5 disassociates SPT5 from *Bscl2* TSS.

We further investigated how PRMT5 regulates the association of SPT5 with *Bscl2* TSS. PRMT5 has been shown to interact with and methylate SPT5.^[^ ^30^ ^]^ We performed Co‐IP experiments in undifferentiated (D0) and differentiated (D6) 3T3‐L1 cells and found that PRMT5 could be pulled down by SPT5 at D6 (Figure 6f). PRMT5 could also be pulled down by SPT5 in 293T cells (Figure S9g, Supporting Information). We also performed proximity ligation assay (PLA) on undifferentiated and differentiated SVF preadipocytes. The PLA showed numerous nuclear puncta in the differentiated adipocyte cells, indicating that PRMT5 physically interact with SPT5 in the nucleus (Figure 6g). Co‐staining of PRMT5 and SPT5 also indicated a nuclear colocalization in LD‐laden adipocytes, but not in preadipocytes (Figure S9h,i, Supporting Information). Similar PLA results were found in differentiated 3T3‐L1 cells, where PRMT5 and SPT5 had strong interaction (Figure S9j, Supporting Information).

To determine if the association between PRMT5 and SPT5 leads to methylation of SPT5, we used a specific antibody that recognizes sDMA (SYM10) to pull down lysates from adipose tissues to check if SPT5 could be detected. Western blotting of total lysates from eWAT of WT and Prmt5^AKO^ mice by SYM10 antibody showed multiple bands whose level was lower in Prmt5^AKO^ lysates (Figure S9k, Supporting Information), suggesting that *Prmt5* KO reduces symmetric demethylated arginine of proteins. We also performed IP with SYM10 antibody and detected a putative SPT5 band between 130 and 170 kDa only in WT but not Prmt5^AKO^ eWAT lysates (Figure S9l, Supporting Information). SYM10 IP followed by SPT5 blotting using the same samples confirmed a robust symmetrically dimethylated SPT5 band in the WT sample, and the intensity of the sDMA SPT5 band was nearly abolished in Prmt5^AKO^ lysates (Figure 6h). These data demonstrated that PRMT5 regulates *Bscl2* expression through methylation of SPT5.

### 2.6 PRMT5 Methylates SREBP1a to Regulate Lipid Metabolism

**Figure 7:**
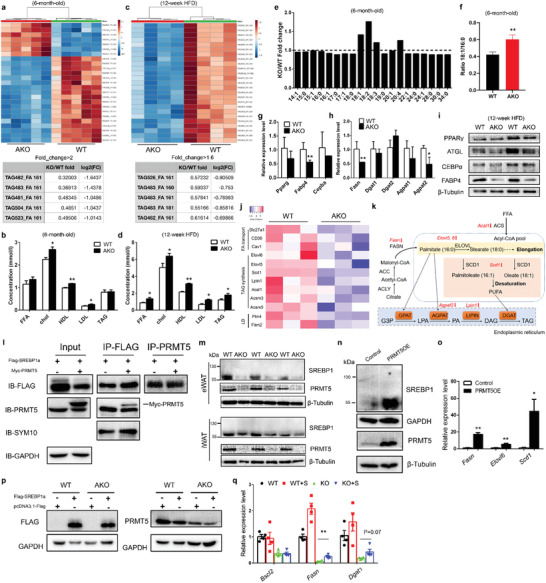
Prmt5 regulates fatty acid metabolic pathways through interacting with SREBP1a. a) Heatmap of top 25 significant changed TAGs from eWAT of WT (Prmt5^flox/flox^) and Prmt5^AKO^ mice (upper) and list of TAGs that were decrease by >2‐fold in Prmt5^AKO^ compared to WT (Prmt5^flox/flox^) eWAT (lower), *n* = 5 pairs male mice of 6‐month‐old. b) Concentration of FFA, cholesterol, HDL, LDL, and TAG from the serum of WT (Prmt5^flox/flox^) and Prmt5^AKO^ mice, *n* = 4 pairs male mice of 6‐month‐old. c) Heatmap of top 25 significant changed TAGs from WT (Prmt5^flox/flox^) and Prmt5^AKO^ mice (upper) and list of TAGs that were decreased by >1.6‐fold in Prmt5^AKO^ compared to WT (Prmt5^flox/flox^) eWAT (lower), after 12‐week of HFD, *n* = 4 pairs of male mice. d) Concentration of FFA, cholesterol, HDL, LDL, and TAG from the serum of WT (Prmt5^flox/flox^) and Prmt5^AKO^ mice, *n* = 3 pairs male mice after 12‐week HFD feeding. e) Fold change of Free Fatty Acids (FFA) in eWAT of WT (Prmt5^flox/flox^) and Prmt5^AKO^ mice. f) Ratio of 18:1/16:0 FFA indicating de novo lipogenesis. e,f) *n* = 5 pairs of 6‐month‐old male mice. g,h) Expression of g) adipogenic and h) lipogenic genes in eWAT from WT (Prmt5^flox/flox^) and Prmt5^AKO^ mice, *n* = 3 pairs of male mice at 6‐month‐old. i) Representative Western blots showing relative protein levels of PPAR*γ*, ATGL, CEBP*α*, FABP4, and *β*‐Tubulin from eWAT of male WT (Prmt5^flox/flox^) and Prmt5^AKO^ mice after 12‐week of HFD. j) Heatmap from RNA‐sequencing data showing relative expression of genes related to Fatty acid transport, TAG synthesis and LD biogenesis in eWAT of WT (Prmt5^flox/flox^) and Prmt5^AKO^ mice, *n* = 3 pairs male mice of 6‐month‐old. k) A diagramatic pathway showing enzymes involved in triacylglycerol (TAG) synthesis, red arrows represent genes down‐regulated in Prmt5^AKO^ eWAT. i) Lysates from 293T cells overexpressing Flag‐SRBEP1a or Flag‐SRBEP1a and Myc‐PRMT were immunoprecipitated (IP) with Flag or PRMT5 antibody and blotted with Flag, PRMT5, SYM10, and GAPDH antibodies. m) Representative Western blots showing relative protein levels of SREBP1, PRMT5, and *β*‐Tubulin from eWAT of male WT (Prmt5^flox/flox^) and Prmt5^AKO^ mice after 12 week of HFD. n) Representative Western blots showing relative protein levels of SREBP1, PRMT5, GAPDH, and *β*‐Tubulin from control and PRMT5‐overexpressed 3T3‐L1 cell line. o) Expression of genes related to TAG synthesis after PRMT5 overexpression, samples were collected from three different wells with three technical repeats for each. p) Representative Western blots showing the overexpression of Flag‐SRBEP1a in SVF preadipocytes from iWAT of 8‐week‐old male WT (Prmt5^flox/flox^) and Prmt5^AKO^ mice after 8 day of differentiation in vitro. q) Expression of *Bscl2*, *Fasn*, and *Dgat1* in SVF preadipocytes from iWAT of 8‐week‐old male WT (Prmt5^flox/flox^) and Prmt5^AKO^ mice with/without Flag‐SRBEP1a overexpression after 8 day of differentiation in vitro, *n* = 3 pairs of mice and cells from each mouse received three individual transfections. Data represent mean ± s.e.m. (*t*‐test: *p < 0.05, **p < 0.01).

The insulin resistance and impaired energy expenditure phenotypes prompted us to examine whether lipid metabolism is affected by *Prmt5* knockout. We performed lipidomics analysis on eWAT from 6‐month‐old Prmt5^AKO^ and WT mice. The analysis identified 191 TAGs whose relative abundance were significantly different, including 137 downregulated and 54 upregulated TAGs in eWAT of Prmt5^AKO^ mice compared to WT mice (Table S1, Supporting Information). Among the top 25 altered TAGs, the downregulated TAGs were mostly TAG of 48–52 carbons, while the up‐regulated were TAG of 52–54 carbons (**Figure** **7**a). The Five TAGs exhibiting fold changes greater than 2 were all downregulated in Prmt5^AKO^ eWAT, and all containing a palmitoleic acid (C16:1) chain (Figure 7a). Analysis of serum lipids shows an opposite upregulation of lipids in Prmt5^AKO^ mice (Figure 7b). The serum cholesterol (chol), high‐density lipoprotein (HDL) and low‐density lipoprotein (LDL) were all significantly increased in Prmt5^AKO^ mice compared with WT mice (Figure 7b). Hence, TAG contents in eWAT were predominantly decreased, associated with increased levels of circulating serum lipids, in the Prmt5^AKO^ mice compared to the WT mice.

We also performed lipidomics on eWAT after mice were fed with HFD for 12 weeks. The relative abundance of 195 TAGs were altered, including 178 downregulated, and 17 upregulated TAGs in Prmt5^AKO^ relative to WT eWAT (Table S1, Supporting Information). All the top 25 significantly changed TAGs were downregulated and were TAG of 48–60 carbons with different levels of unsaturation (Figure 7c). Five TAGs were found to have fold changes higher than 1.6 and were all decreased in Prmt5^AKO^ relative to WT eWAT (Figure 7c). Interestingly, TG‐483_FA‐161 and TG‐482_FA‐161 were consistently ranked as top downregulated TAGs in both normal diet and HFD treated in Prmt5^AKO^ mice (Figure 7a,c). In contrast, free fatty acids (FFA), chol, HDL, LDL, and TAG were all significantly increased in the serum of Prmt5^AKO^ mice compared with WT mice after HFD treatment (Figure 7d). In addition, liver from Prmt5^AKO^ mice at 10‐month‐old and after HFD were larger due to fatty infiltration and steatosis (Figure S10a–d, Supporting Information). These data indicate that HFD exacerbates the lipid metabolism deficiency in Prmt5^AKO^ mice.

We also found that the relative abundances of polyunsaturated fatty acids (PUFAs) and C18:1 monounsaturated fatty acid (MUFA) were increased in KO eWAT, while other MUFAs and saturated fatty acids were reduced (Figure 7e). The ratio of FFA C18:1/C16:0, as an indicator of defective *de novo* lipid synthesis, was significantly increased in KO eWAT (Figure 7f). We further investigated how PRMT5 regulates lipid metabolism by profiling expression of genes involved in adipogenesis, lipogenesis, and lipolysis. The mRNA levels of *Fabp4*, *Fasn*, and *Agpat2* were significantly lower in Prmt5^AKO^ compared to WT eWAT (Figure 7g,h). The protein levels of ATGL, which catalyzes the initial step in triglyceride hydrolysis,^[^ ^31^ ^]^ and FABP4 were also lower in eWAT of Prmt5^AKO^ than WT mice after fed with HFD for 12 weeks (Figure 7i). We also performed unbiased RNA‐sequencing analysis on eWAT isolated from 6‐month‐old WT and Prmt5^AKO^ mice. Many genes related to FA transport (*Slc27a1*, *CD36*, *Caveolin1*), TAG synthesis (*Elovl6*, *Elovl5*, *Scd1*, *Lipin1*, *Acsl1*, *Acsm3*, *Acsm5*), and LD biogenesis (*Perilipin4*, *Fitm2*) were decreased in Prmt5^AKO^ mice (Figure 7j). These results together indicate that PRMT5 plays a role in regulating expression of key lipogenic genes (Figure 7k). Collectively, the gene expression profiling results suggest that *Prmt5* knockout causes defects in fatty acid metabolic pathways in WAT, leading to elevated serum lipids and hepatic steatosis.

To understand how PRMT5 regulates TAG synthesis, we intersected recently reported PRMT5 substrates and lipogenic genes.^[^ ^26^ ^]^ This analysis identified SREBP1a, which has been reported to be methylated and stabilized by PRMT5.^[^ ^32^ ^]^ We performed Co‐IP experiments in 293T cells and found that methylated SREBP1a could be pulled down by PRMT5 (Figure 7l). Conversely, PRMT5 could also be pulled down by SREBP1a (Figure 7l). The protein levels of SREBP1 in WAT, especially eWAT, were dramatically decreased in Prmt5^AKO^ than WT mice, (Figure 7m). Consistently, SREBP1 protein level in differentiated SVF cells was also lower in Prmt5^AKO^ cells compared to WT cell (Figure S11a, Supporting Information). Conversely, *Prmt5* overexpression elevated the protein level of SREBP1, accompanied by increased mRNA levels of SREBP1 target genes *Fasn, Elovl6*, and *Scd1* (Figure 7n,o). We then overexpressed *SREBP1a* in cultured SVF cells from Prmt5^AKO^ and WT mice (Figure 7p, Figure S11b, Supporting Information). *SREBP1a* overexpression upregulated mRNA level of *Fasn* in WT cells and partially restored *Fasn* expression in Prmt5^AKO^ adipocytes (Figure 7q). However, *SREBP1a* overexpression had no effects on *Bscl2* expression and LD size in Prmt5^AKO^ cells (Figure 7q and Figure S11c, Supporting Information). These results indicate that PRMT5 regulates de novo lipogenesis in WAT at least partially through SREBP1a.

**Figure 8:**
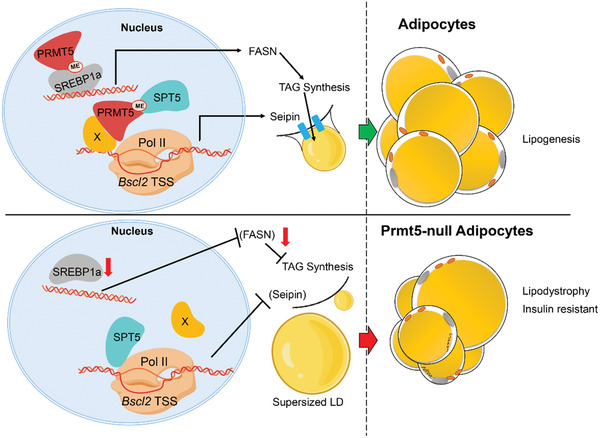
A model depicting PRMT5's role in regulating lipogenesis. PRMT5 regulates *Bscl2* expression by methylating SPT5 during Lipid droplet formation. Prmt5 also methylates SREBP1a to promote TAG synthesis. X: unknown factors connecting PRMT5 to *Bscl2* TSS.

Together, we propose a model in which PRMT5 expression in newly differentiated adipocytes methylates SPT5 and disassociates the transcriptional pausing factor from *Bscl2* TSS. *Bscl2* transcription leads to increased protein levels of Seipin that is critical for the biogenesis of LDs (**Figure** **8**. Meanwhile, PRMT5 methylates and stabilizes SREBP1a, leading to upregulation of SREBP‐target genes to promote *de novo* lipogenesis and TAG deposition into the LD (Figure 8).

## 3 Discussion

Our study demonstrates a previously unrecognized role of PRMT5 in regulating the fatty acid metabolism and LD formation in WAT, thus affecting whole body metabolism and insulin sensitivity. Adipocyte‐specific deletion of *Prmt5* leads to the age‐dependent loss of WAT mass with smaller white adipocytes. In addition, the reduced WAT mass and body weight loss is exacerbated by HFD. Notably, the small adipocytes in Prmt5^AKO^ mice do not contribute to an improvement in insulin sensitivity as expected.^[^ ^33^ ^]^ On the contrary, *Prmt5* KO causes a progressive insulin resistance by disrupting fatty acid metabolism pathways and Seipin‐dependent LD formation in WAT. Furthermore, arginine methylation of SPT5 by PRMT5 in adipocytes is critical for the disassociation of SPT5 from *Bscl2* TSS. As previous studies have implicated SPT5 in transcriptional elongation and pausing,^[^ ^34^ ^]^ the dissociation of SPT5 from *Bscl2* TSS presumably resumes POL2‐mediated transcription of *Bscl2*. PRMT5 also interacts with and methylates SREBP1a, which subsequently enhances the transcription of TAG synthesis related genes, such as *Fasn*. The present study suggests that LD formation through the PRMT5‐SPT5‐*Bscl2* pathway is critical to ensure proper biogenesis of LDs and the TAG synthesis through PRMT5‐SREBP1a pathway, consequently protecting mice from diet/age‐induced metabolic disorder and insulin resistance in vivo. Future studies dissecting the role PRMT5 in human adipose tissue will reveal if a similar regulatory network exist in human cells.

We used an adipocyte‐specific *Adipoq‐Cre* to drive the deletion of *Prmt5* in adipose tissues.^[^ ^35^ ^]^ In general, the observed phenotypes are somewhat similar to those of adipocyte specific PPAR*γ* or mTOR deletion in terms of lipodystrophy.^[^ ^36^ ^]^ However, in our Prmt5^AKO^ mice the lipodystrophy phenotype was more specific to WAT, especially eWAT and asWAT. The WAT depot‐specific phenotype explains why female mice had a stronger phenotype than males, as female mice typically have a higher percentage of body fat and have increased adipocyte precursor cells and mature adipocytes in gonadal fat (eWAT) after HFD treatment.^[^ ^37^ ^]^ Depot, age, and gender specific effects of various other genes has also been reported, for example Cited4 has distinct roles in regulating thermogenic gene expression in female and male mice, and different fat depots.^[^ ^38^ ^]^ Previous in vitro studies showed that knockdown of *Prmt5* in 3T3‐L1 cells inhibits adipogenic differentiation and adipogenic gene expression,^[^ ^17^ ^]^ through a mechanism involving Prmt5 facilitated enhancer–promoter looping to promote expression of Ppar*γ*2.^[^ ^18^ ^]^ However, we did not observe a very significant decrease in *Pparγ* expression in *Prmt5*‐null adipocytes. This discrepancy may be explained by the Adipoq‐Cre model that we used, which drives deletion of Prmt5 in post‐differentiation adipocytes and thus bypass the effect on adipogenesis. This discrepancy suggests different roles of Prmt5 in preadipocytes and mature adipocytes. In our model, we observed a more specific reduction in genes related to fatty acid metabolic pathways. Combining the observation that PRMT5 is expressed at higher level in the late phase of differentiation and its immunofluorescence is more abundant in differentiated adipocytes, these results indicate that PRMT5 plays a more important role in in mature adipocytes than in preadipocytes. The interesting observation that the old Prmt5^AKO^ mice were insulin resistant but glucose tolerant may be due to compensatory glucose disposal by the skeletal muscle, as the lean mass of Prmt5^AKO^ mice was greater than WT mice at 10‐month old. We also found that RER of Prmt5^AKO^ mice were higher at 10‐month old and after HFD treatment, indicating a fuel source switch from fat to carbohydrate in response to *Prmt5* knockout. Thus, future studies evaluating the systematic glucose uptake would be warranted to directly address the possibility.

We found that the expression of LD biogenesis related genes is reduced in Prmt5^AKO^ mice. Tightly controlled LD formation and expansion from ER is important for the lipid homeostasis and adipogenesis. We also observed that the LD structure is disrupted in *Prmt5* knockout adipocytes and ER structure defects in Prmt5^AKO^ eWAT. Specifically, the *Prmt5*‐null adipocytes phenocopy one of the most prominent feature of Seipin‐deficient cells in the formation of “supersized” LDs.^[^ ^39^ ^]^ Furthermore, Prmt5^AKO^ mice also phenocopy the lipodystrophy and lipidomic profiling phenotypes of the adipose‐specific Seipin KO (ASKO) mice and in vitro behavior of Seipin‐null adipocytes.^[^ ^25^, ^40^ ^]^ Consistently, we showed that knockout and pharmacological inhibition of PRMT5 both suppressed *Bscl2* expression. Overexpression of Seipin partially restored the sizes of LDs further support PRMT5 acting through Seipin to regulate LD homeostasis. Interestingly, we did not find any obvious defects in BAT of Prmt5^AKO^ mice. This phenotype was different from UCP‐1^Cre^ mice mediated *Bscl2* deletion in BAT, which shows reduced BAT mass and supersized unilocular LDs in BAT.^[^ ^41^ ^]^ The strong phenotypes in WAT, especially eWAT, of Prmt5^AKO^ mice may have masked any subtle phenotypes in BAT. Future study using UCP1^Cre^ driven *Prmt5* knockout specifically in brown adipocytes will elucidate the function of PRMT5 in BAT.

We used ChIP‐sequencing assay to demonstrate that PRMT5 binds to the TSS region of *Bscl2* after differentiation, supporting a direct connection between PRMT5 and *Bscl2* expression. As PRMT5 typically functions through methylation of its substrates, either histone proteins or non‐histone proteins, we speculated that PRMT5 should most likely regulate *Bscl2* expression by interacting with another protein. Previous work demonstrates that PRMT5 methylates SPT5 in the region containing the KOW domains and arginine methylation of SPT5 inhibits its ability to associate with promoter‐bound POL2.^[^ ^30^ ^]^ Consistent with this notion, by analyzing the ChIP‐seq data in mES cells and in mouse eWAT,^[^ ^27^, ^28^ ^]^ we identified a common binding region at *Bscl2* TSS by SPT5, POL2, and PRMT5. Interestingly, binding affinity of SPT5 to *Bscl2* TSS is dramatically decreased after differentiation or *Prmt5* overexpression, suggesting that PRMT5 negatively regulate SPT5 binding to the *Bscl2* TSS. SPT5 is a subunit of the DSIF (DRB Sensitivity Inducing Factor) complex which can either negatively or positively affect transcription by POL2. DSIF interacts with negative elongation factor (NELF) to promote the pausing of POL2 at some genes.^[^ ^42^ ^]^ In addition, PRMT5 methyltransferase activity also requires the interaction with its cofactors such as MEP50 or BRG1 to form a complex.^[^ ^43^ ^]^ In our model, we speculated that SPT5 acts as a transcriptional pausing factor in preadipocytes and arginine methylation by PRMT5 after adipogenic differentiation releases SPT5 from *Bscl2* TSS to promote its transcription. However, there are factors other than PRMT5‐SPT5‐POL2 in this complex that are required for maintaining the continuous *Bscl2* elongation despite SPT5's dissociation, which needs to be evaluated in further study. PRMT5 preserves a substrate preference to methylate arginine at Arg‐Gly (RG) and Gly‐Arg‐Gly (GRG).^[^ ^44^ ^]^ Pharmacological and genetic inhibition of PRMT5 demonstrate that 681R and 696R of human SPT5 are critical arginine residues methylated by PRMT5.^[^ ^26^ ^]^ The 696R (R690 in *M. Musculus* Spt5) is followed by a Gly, forming a featured PRMT5 targeting RG motif, and this RG motif is conserved across the vertebrates. In addition, the R696 is located closely to the KOW5 domain of SPT5, which is critical for its interaction with POL2.^[^ ^30^ ^]^ Thus, the arginine methylation of SPT5 at 696R by PRMT5 is probably a specific conserved signal that initiates the LD biogenesis. This is the first example of an evolutionarily conserved genetic regulator of *Bscl2* expression. Congenital generalized lipodystrophy type 2, also known as Berardinelli‐Seip congenital lipodystrophy type 2 (BSCL2), is a disease with a wide spectrum of inherited and acquired syndromes characterized by lipodystrophy, hypertriglyceridemia, and insulin resistance.^[^ ^45^ ^]^ Mutations of four genes have been linked to BSCL2, with BSCL2 mutations representing the most severe form of human BSCL2.^[^ ^46^ ^]^ Our findings suggest that dysregulation of PRMT5 may also contribute to the development of BSCL2.

Despite the similar lipodystrophy phenotype in Prmt5^AKO^ and Bscl2^AKO^ mice, the hepatic steatosis, insulin resistance, and lowever energy expenditure of Prmt5^AKO^ mice were not observed in Bscl2^AKO^ mice.^[^ ^25^ ^]^ The distinct phenotype may be explained by additional targets of PRMT5 besides Seipin. In this scenario, we found that genes (*Fasn, Scd1*, and *Elovl6*) in the TAG synthesis pathway were inhibited by Prmt5^AKO^. FASN is a key enzyme that catalyzes de novo synthesis of palmitate, and SCD1 converts saturated fatty acids into monounsaturated fatty acids.^[^ ^47^ ^]^ ELOVL6 is identified as an SREBP target gene that catalyzes elongation of saturated and monounsaturated acyl‐CoAs with 12–16 carbons.^[^ ^48^ ^]^ The expression of all these three genes were down‐regulated in *Prmt5*‐null WAT and elevated by *Prmt5* overexpression, indicating that PRMT5 also regulates TAG synthesis. We further show that PRMT5 regulate lipogenic genes through interacting with and methylating SREBP1a. Consistent with this finding, PRMT5 has been reported to methylate SREBP1a at R321 and this modification in turn stabilizes the cleaved nuclear form of SREBP1a, thus upregulating lipogenic gene expression and promoting de novo lipogenesis.^[^ ^32^, ^49^ ^]^ Interestingly, the LD size as well as the expression of *Bscl2* were not rescued by SREBP1a, suggesting that PRMT5‐SREBP1a pathway mainly regulates fatty acid synthesis without affecting LD biogenesis.

The development of T2D is accompanied by impaired systemic glucose tolerance. By analyzing previously published data,^[^ ^21^ ^]^ we found that *PRMT5* expression in WAT is positively associated with the glucose tolerance and have lowest expression in T2D. As patients with T2D are usually linked with overweight and obese, we examined if PRMT5 expression is corrected to body weight. Through analyzing a published dataset,^[^ ^20^ ^]^ we could not establish a correlation between *PRMT5* in WAT and body weight status. A study following up 2625 T2D participants for over 10 years showed that 12% diabetic patients with normal body weight have almost twice higher overall mortality rate.^[^ ^50^ ^]^ We found that *PRMT5* expression in WAT is positively correlated with the body weight status in T2D patients by analyzing a published dataset.^[^ ^21^ ^]^ In our mouse model, especially after HFD treatment, *Prmt5* deficiency also leads to a lean but more diabetic phenotype. As SPT5 also bind at human *BSCL2* TSS region, a similar functional requirement for the PRMT5‐SPT5‐Seipin pathway in maintaining the homeostasis of WAT may exist in humans. Based on these lines of evidence, it is important to examine how dysregulation of PRMT5 occurs and contributes to the insulin resistant in animal models and non‐obese T2D patients.

## 4 Experimental Section

##### Mice and Animal Care

The *Adipoq‐Cre* (stock #01 0803) mice were obtained from Jackson Laboratory. The frozen sperms from mice harboring *Prmt5^tm2c(EUCOMM)wtsi^* were purchased from Wellcome Trust Sanger Institute. The in vitro fertilization, embryo development and implantation in female C57BL/6 were performed in the Purdue University Transgenic and Genomic Editing Facility. PCR‐based genotyping was performed to screen for *Prmt5^f/f^* mice. Request for *Prmt5^f/f^* mice should be directed to Dr. Chang‐Deng Hu at hu1@purdue.edu. The *Adipoq‐Cre* and *Prmt5^f/f^* mice were intercrossed for several generations to generate the Prmt5^AKO^ mice. Mice were genotyped by PCR of ear DNA using primers listed in Table 1, Supporting Information. The genotypes of experimental KO and associated control animals were as follows: Prmt5^AKO^ (*Adipoq‐Cre*:: *Prmt^f/f^*) and WT (*Prmt^f/f^*). Mice were housed in the animal facility with free access to water and standard rodent chow food (control diet) or HFD (TD.06414 Harlan). Food intake assay was calculated by measuring food consumption weekly, the average food consumption of two mice per day from the same cage was considered as one group. Unless otherwise indicated, 8 week, 3–10 month old male mice were analyzed in this study. Mouse maintenance and experimental use were performed according to protocols approved by the Purdue Animal Care and Use Committee (under animal protocol number 1112000440).

##### Indirect Calorimetry Study

Oxygen consumption (VO_2_), carbon dioxide production (VCO_2_), respiratory exchange ratios (RER), and heat production were measured by using indirect calorimetry system (Oxymax, Columbus Instruments), installed under a constant environmental temperature (22 °C) and a 12 h light (06:00–18:00 h), 12 h dark cycle (18:00–06:00 h). Mice in each chamber had free access to food (chow diet or HFD) and water. The raw data were normalized by body muscle mass and the histograms of day (06:00–18:00 h) and night (18:00–06:00 h) values were the mean value of all points measured during the 12 h period.

##### Hematoxylin–Eosin and Immunofluorescence Staining

Adipose tissues from the WT and Prmt5^AKO^ mice were fixed in 4% PFA for 24 h at room temperature. Then the tissues were embedded into paraffin, blocked, and cut at 6 mm for H&E staining. For H&E staining, the sections were deparaffinized, rehydrated, and the nuclei stained with hematoxylin for 15 min. Sections were then rinsed in running tap water and stained with eosin for 3 min, dehydrated, and mounted. Images were captured using a Leica DM 6000B fluorescent microscope.

Immunofluorescence was performed in cultured SVF preadipocytes and 3T3‐L1 cells. Briefly, samples were fixed in 4% paraformaldehyde for 5 min and then permeabilized and blocked in PBS containing 5% goat serum, 2% bovine serum albumin (BSA), 0.2% Triton X‐100, and 0.1% sodium azide for 1h. Samples were subsequently incubated with diluted primary antibodies (Prmt5, Millipore: 07–405, 1:500, and Spt5, sc‐133217, 1:500) overnight at 4 °C. After washing with PBS, the samples were incubated with secondary antibodies and DAPI for 1 h at room temperature. Fluorescent images were captured with a CoolSnap HQ charge coupled‐device camera (Photometrics) by using a Leica DM6000 microscope (Leica).

##### Blood Glucose Measurements

For GTT, mice were given i.p. injection of 100 mg ml^−1^ d‐glucose (2 g kg^−1^ body weight on mice with control diet, 0.5 g kg^−1^ body weight on HFD) after overnight fasting for 14 h, and tail blood glucose concentrations were measured by a glucometer (Accu‐Check Active, Roche) every 30min after injection continuously for 2 h. For ITT, mice were fasted for 5 h before i.p. administration of human insulin (Santa Cruz) (0.75U per kg body weight), and tail blood glucose concentrations were monitored. For both GTT and ITT, mice were caged with blinded cage number and random orders.

##### Cell Culture

Primary WAT SVF preadipocytes were isolated using collagenase digestion and followed by density separation. Briefly, the inguinal or epidydimal white adipose were minced and digested in 1.25 mg mL^−1^ collagenase type I at 37 °C for 45–60 min. The digestion was terminated with DMEM containing 10% FBS, and filtered through 70 µm filters to remove undigested tissues. Cells were then centrifuged at 1700 rpm for 5 min to separate the SVF preadipocytes in the sediment and lipid‐containing adipocytes in the floating layer. The freshly isolated SVF cells were seeded and cultured in growth medium containing DMEM, 20% FBS, 1% penicillin/streptomycin (P/S) at 37 °C with 5% CO_2_ for 3 days, followed by changing fresh medium every 2 days. The 3T3‐L1 (ATCC) and HEK293T (ATCC) cell line were cultured in DMEM with 10% FBS. For adipogenic differentiation, the cells were induced with induction medium contains DMEM, 10% FBS, 2.85 mm insulin, 0.3 mm dexamethasone (DEXA), 1 mm rosiglitazone, and 0.63mm 3‐isobutyl‐methylxanthine (IBMX) for 4 days on confluence and then differentiated in differentiation medium contains DMEM, 10% FBS, 200nm insulin, and 10 nm T3. To avoid the effect of cell density on adipogenic differentiation, cells were induced to differentiate when they reach 100% confluence. pcDNA3.1‐2xFLAG‐SREBP‐1a plasmid was a gift from Timothy Osborne (Addgene plasmid # 26 801; http://n2t.net/addgene:26801; RRID:Addgene_26 801).^[^ ^51^ ^]^ For transfection, SVF preadipocytes from WT and KO mice were seeded into six‐well plate and allowed to grow until 90% confluency. Then each well was transfected with 1mg of pcDNA3.1‐2xFLAG‐SREBP‐1a or pcDNA3.1 plasmid together with the induction medium. Samples were collected 6 days after transfection for further protein and RNA analysis. Each transfection was performed in three biological and three technical repeats (three pairs of mice).

##### Adipocyte OCR Measurement

Primary SVF preadipocytes from inguinal WAT were isolated and cultured for 2 days before being plated in XF cell culture microplates (Seahorse Bioscience). 30 000 cells were seeded in each well, and after differentiated 8 days, cultured adipocytes were washed twice and pre‐incubated in XF medium (supplemented with 25 mm glucose, 2 mm glutamine, and 1mm pyruvate) for 1 h at 37 °C without CO2. The OCR was measured using the XF Extracellular Flux Analyzer (Seahorse Biosciences). Oligomycin (2 mm), FCCP (2 mm), and Antimycin A and Rotenone (0.5 mm) were preloaded into cartridges and injected into XF wells in succession. OCR was calculated as a function of time (picomoles per minute).

##### Total RNA Extraction and Real‐Time PCR

Total RNA was extracted from cells or tissues using trizol reagent according to the manufacturer's instructions. The purity and concentration of total RNA were measured by a spectrophotometer (Nanodrop 3000, Thermo Fisher) at 260 and 280 nm. Ratios of absorption (260/280 nm) of all samples were ≈2.0. Then 3 µg of total RNA were reversed transcribed using random primers and MMLV reverse transcriptase. Real‐time PCR was carried out with a Roche Lightcycler 480 PCR System using SYBR Green Master Mix and gene‐specific primers. Primer sequences are listed in Table S2, Supporting Information. The 2^−ΔΔCT^ method was used to analyze the relative changes in gene expression normalized against mouse *β*‐Actin as internal control.

##### Protein Extraction and Western Blot Analysis

Protein was extracted from homogenized muscle tissue or muscle cells with RIPA buffer (150 mm NaCl, 1% NP‐40, 0.5% sodium Deoxycholate, 0.1% SDS50 mm Tris‐HCl, pH 8.0) that contained a protease inhibitor cocktail (Sigma) and phosphatase inhibitors NaF and Na_3_VO_4_. Protein concentration was measured using the BCA protein quantification kit (Pierce). Equal amounts of each protein sample were loaded for electrophoresis (Bio‐Rad). Protein concentrations were determined using Pierce BCA Protein Assay Reagent (Pierce Biotechnology). Proteins were separated by SDS–PAGE, transferred to a polyvinylidene fluoride membrane (Millipore Corporation), blocking in 5% fat‐free milk for 1 h at room temperature (RT), then incubated with primary antibodies in 5% milk overnight at 4 °C. The Prmt5 antibody was from Millipore (07‐405, 1:1000), *β*‐Tubulin was from Abcam (ab6046, 1:5000), Atgl was from Cell Signaling Technology (2439, 1:1000), *Srebp1* was from Invitrogen (MA5‐16124, 1:1000), other antibodies were from Santa Cruz Biotechnology (Santa Cruz), including Ppar*γ* (sc‐7273, 1:1000), Glut4 (sc‐7938, 1:500), Fabp4 (sc‐271529, 1:1000), C/EBP*α* (sc‐61,1:1000), Spt5 (sc‐133217, 1:200), and GAPDH (sc‐32233, 1:1000). The horseradish peroxidase (HRP)‐conjugated secondary antibody (anti‐rabbit IgG, 111‐035‐003 or anti‐mouse IgG; 115‐035‐003, Jackson ImmunoResearch) were diluted 1:10000 and incubated at RT for 1 h. Immunodetection was performed using enhanced chemiluminescence Western blotting substrate (Santa Cruz) and detected with FluorChem R System (ProteinSimple). Results shown in the Figure are representative results from at least three independent experiments.

##### RNA‐Seq Analysis

Total RNA was extracted from eWAT of 6 month old WT and KO mice, and subjected to RNA‐seq analysis performed by Novel Bioinformatics Co., Ltd. (https://en.novogene.com/). Briefly, RNA quality analysis was checked by Agarose Gel Electrophoresis and Agilent 2100. A complementary DNA library was then constructed using mRNA enriched by anti‐polyA beads, and sequencing was performed according to the Illumina HiSeq standard protocol. Raw reads from RNA‐seq libraries are filtered to remove reads containing adapters or reads of low quality. After filtering, statistics analysis of data production and quality was performed to confirm the sequencing quality. Reference genome and gene annotation files were downloaded from a genome website browser (NCBI/UCSC/Ensembl). TopHat2 was used for mapping the filtered reads to the reference genome. For the quantification of gene expression level, HTSeq V0.6.1 was used to analyze the read numbers mapped for each gene. The RPKM of each gene was calculated based on the gene read counts mapped to genes or exons. A differential expression analysis was performed using the DESeq R package (1.10.1) with the threshold of significance set as p‐adjusted <0.05. Heatmap was made by an online tool (http://heatmapper.ca/) based on the Log_2_RPKM.^[^ ^52^ ^]^

##### Lipidomics

For MS analysis, eWAT samples (50mg per mouse) from WT and Prmt5^AKO^ mice at 4–6 months old or after 12 weeks of HFD were sent to the Metabolite Profiling Facility at Purdue University for MRM‐profiling. Lipids were extracted using the Bligh and Dyer extraction method.^[^ ^53^ ^]^ In brief, samples were transferred to a 2 mL vial with inert 1.4 mm ceramic (zirconium oxide) beads (Precellys CK 14, Bertin Corp, part # P000912‐LYSK0A), and 500 µL of ultrapure water was added to homogenize the sample using the Precellys tissue homogenizer (Bertin Corp, Rockville, MD, US) at three cycles of 6200 rpm for 20 s. Next, 200 µL of homogenized tissue was transferred to a new microtube and mixed with 250 µL of chloroform and 450 µL of methanol. This solution was incubated at room temperature for 15 min. After that, 250 µL of chloroform and 250 µL of water were added and the sample was centrifuged for 10 min at 16 000 × *g*, forming a two‐phase solution where the bottom phase contained the lipids (organic phase). The organic phase was transferred to a new tube and dried using a speedvac centrifuge (Savant Speedvac, Thermo Scientific Inc., San Jose, CA, US), and samples were stored at −80 °C until mass spectrometry analysis.

Targeted lipid profiling was performed using discovery MRM‐profiling methods and instrumentation as described.^[^ ^54^ ^]^ Specifically, for sample preparation, dried lipid extracts were diluted in 500 µL of methanol/chloroform 3:1 (v/v) (stock solution). The stock solution was further diluted 50× in injection solvent (aceto‐ nitrile/methanol/ammonium acetate 300 mm 3:6.65:0.35 (v/v)) and and 8µL of this solution was used for the profiling analysis of each lipid class using a micro‐autosampler (G1377A) to the ESI source of an Agilent 6410 triple quadrupole mass spectrometer (Agilent Technologies, Santa Clara, CA, USA). A capillary pump was connected to the autosampler and operated at a flow rate of 7 µL min^−1^ and pressure of 150 bar. Capillary voltage on the instrument was 3.5–5 kV and the gas flow 5.1 L min^−1^ at 300 °C.

For the MS analysis, relative amounts of ion abundances were used for statistics. Values of ion intensities for each of the MRMs monitored were normalized by total ion intensity of all MRMs in the method for a given sample. Further statistical analysis was then performed using Metaboanalyst 4.0 software (https://www.metaboanalyst.ca). Uploaded data was auto‐scaled and volcano plots (Fold change threshold 2, P value threshold 0.05), principal component analyses (PCAs), and heatmaps were plotted. Fold change was calculated by dividing values of ion intensities for each of the MRMs measured in each sample by the ion intensity of the corresponding MRM in the blank. Average fold change was graphed for significantly different lipid species using GraphPad Prism Software Version 8.0.1. Significant changes between groups were analyzed by students’ *t*‐test and *p* values < 0.05 were considered statistically significant.

##### Dual‐Luciferase Reporter Assay

HEK293A cells were seeded into 24‐well plates 1 day before Lipofectamine 2000‐mediated transfection. The pGL3‐Bscl2‐1kb promoter luciferase plasmid was generated by cloning the 1kb promoter region of *Bscl2* into pGL3‐Basic plasmid. The positive clone was confirmed by sequencing. For transfection of each well, 80 ng Renilla plasmid, 250 ng pGL3‐Bscl2‐1kb (or pGL3‐Basic plasmid as control) and 500 ng pcDNA‐Myc‐Prmt5 plasmid were co‐transfected following the manufacturer's instructions. After transfected for 48 h, cells were harvested and analyzed with the Dual‐Luciferase Reporter Assay System (Promega).

##### Transmission Electron Microscopy

The eWAT from 6, 10 month old, and 12 week HFD treated WT and KO mice were cut into 1 × 2 mm blocks in a 6 cm culture dish immediately after euthanizing the mice, and then fixed in 2.5% glutaraldehyde for 30 min. The blocks were continued to be fixed in 2.5% glutaraldehyde for 1 h, followed by fixation in 2% osmium tetroxide for 1 h. All the fixatives were made with 0.1 m cacodylate buffer. After washing, the blocks were dehydrated in a graded ethanol series and then embedded in Epon Generic Resin. The sections with a thickness of about 90 nm were prepared with uranyl acetate and lead citrate stain and examined with a transmission electron microscope (Gatan Digital microscopy).

##### Oil Red O Staining

Cultured cells were washed with PBS and fixed with 4% paraformaldehyde (PFA) for 15 min at RT. Then the cells were stained using the Oil red O working solutions (6 mL 5 g L^−1^ Oil red O stock solution in isopropanol, 4 mL ddH_2_O, filtered for three times) for 30 min. After staining, the cells were washed with 60% isopropanol and pictured.

##### Adenovirus and Retrovirus Generation

The blank adenovirus (pAd‐GFP) and adenovirus with Bscl2 insertion (pAd‐Seipin‐GFP) were gifts from Dr. Weiqin Chen from Medical College of Georgia at Augusta University, Augusta, Georgia, USA. For retroviral overexpression of Prmt5, pBABE‐puro‐Prmt5, pCL‐Eco, and pCMV‐VSV‐G plasmids were co‐transfected into HEK‐293T cells seeded in six‐well plates. After 48 h, the retrovirus was collected and 3T3‐L1 cell line (20%–30% confluent) was infected by control or Prmt5‐OE retrovirus, and then the stable expressing cell were selected by puromycin (1 µg mL^−1^).

##### DNA Sequencing Following Chromatin Immunoprecipitation

Chromatin immunoprecipitation (ChIP) was performed as previously described^[^ ^55^ ^]^ with some modifications. Briefly, undifferentiated (D0) and differentiated (D1) 3T3‐L1 cells were cross‐linked with 1% formaldehyde (Ted Pella Inc., Redding, CA) for 10 min at room temperature. After quenching of the formaldehyde with 125 mm glycine for 5 min, fixed cells were washed twice with ice‐cold phosphate‐buffered saline (PBS) supplemented with protease cocktail inhibitor (PCI) (Thermo Scientific, A32965) and resuspended in cell lysis buffer (10 mm Tris HCl, 10 mm NaCl, 0.5% NP‐40, and PCI, pH = 7.5), then were incubated on ice for 10 min. The nuclei were pelleted, washed with MNase digestion buffer (20 mm Tris HCl, 15 mm NaCl, 60 mm KCl, 1 mm CaCl2, and PCI, pH = 7.5), and incubated for 20 min at 37 °C in the presence of 2000 gel units of micrococcal nuclease (NEB, M0247S) per mL of MNase digestion buffer. The reaction was stopped by adding equal volume sonication buffer (100 mm Tris HCl 20 mm EDTA, 200 mm NaCl, 0.2% sodium deoxycholate, 2% Triton X‐100, and PCI, pH = 8.1,). Samples were sonicated for 10–12 min (high intensity, 30s on/30s off) in a Bioruptor Pico Sonicator (Diagenode, Denville, NJ) at 4 °C, and centrifuged at 21000 × *g* for 5 min. The length of the fragmented chromatin was between 200 and 500 bp as analyzed on agarose gels. Chromatin concentrations were measured using a Qubit 3 fluorometer (Invitrogen). 5 µg chromatin was subjected to immunoprecipitation with rabbit polyclonal Prmt5 antibody (Epigentek, A‐3005‐100) or with anti‐IgG as a negative control at 4 °C overnight. Immunocomplexes were recovered by incubation with protein A‐agarose magnetic beads (Invitrogen) for 3–4 h at 4 °C. Sequential washes of 5 min each were performed with ChIP buffer (50 mm Tris, 10 mm EDTA, 100 mm NaCl, 1% Triton X‐100, and 0.1% sodium deoxycholate, pH = 8.1), ChIP buffer with 0.5 m NaCl, Tris/LiCl buffer (10 mm Tris, 1 mm EDTA, 0.25 M LiCl2, 0.5% NP‐40, and 0.5% sodium deoxycholate, pH = 8.1) and Tris/EDTA buffer (50 mm Tris and 10 mm EDTA, ph = 8.0). Immune complexes were eluted from the Protein A magnetic beads by incubation in 100 µL of elution buffer (10 mm Tris HCl, 10 mm EDTA, 150 mm NaCl, 1% SDS, and 5 mm DTT, ph = 8.0) for 30 min at 65 °C. Eluates were then reverse cross‐linked overnight at 65 °C. Following this, samples were treated with 1µL RNase A (10 mg mL^−1^, Thermo Scientific, EN0531) for 1 h at 37 °C and 1 µL of Proteinase K (20 mg mL^−1^, Invitrogen, AM2548) for 2 h at 37 °C. DNA was purified using a ChIP DNA Clean and Concentrator kit (Zymo Research, Irvine, CA).

ChIP was performed in duplicates and sequencing libraries were prepared using the ThruPLEX DNA‐seq kits (Rubicon Genomics). Libraries were prepared with 0.1–33.2 ng of ChIP DNA or input and individually barcoded. qPCR library amplification was performed for five to nine cycles. Libraries were sequenced using an Illumina NextSeq High Output instrument with paired‐end 75‐bp reads. Read depth ranged between 37 and 52 million reads per replicate. Fastq files were aligned to the 10 mm reference genome with bowtie2.^[^ ^56^ ^]^ Reads with a mapq score of 20 or greater were retained using Samtools.^[^ ^57^ ^]^ PCR duplicates were filtered out using picard. Peak calling was performed using macs2.^[^ ^58^ ^]^

##### ChIP‐qPCR Assay

Culture blank and PRMT5‐overexpressed 3T3‐L1 cells or SVF preadipocytes from the WT and KO mice were crosslinked using fixed with 3.7% formaldehyde for 10 min at room temperature with shaking, followed by the addition of 125 mm glycine for 5 min at room temperature, after which samples were washed twice with cold PBS and placed in ChIP cell lysis buffer containing 20 mm Tris, 0.1% SDS, 1% Triton‐100, 150 mm NaCl, 1 Mm EDTA and protease inhibitor on ice for 10 min. After centrifugation, the nuclei were resuspended in muclear lysis buffer and sonicated. Polyacrylamide gel electrophoresis was used to confirm the size of DNA fragment. Then the lysis was diluted for IP with the indicated antibodies (anti‐SPT5/PRMT5, 1:50) and incubation at 4 °C overnight. The immunoprecipitants were eluted and reverse crosslinked overnight at 65 °C. DNA fragments were purified using the Using Phenol–Chloroform method and quantitative PCR was performed. Primer sequences are listed in Table S2, Supporting Information. Each ChIP‐qPCR were performed in three biological and two technical repeats.

##### Co‐IP Assay

HEK293T cells were transfected with pcDNA3.1‐2xFLAG‐SREBP‐1a or pcDNA3.1‐2xFLAG‐SREBP‐1a +pcDNA‐Myc‐Prm5, and total protein was extracted after 48 h. The lysate was precleared with protein A/G agarose at 4 °C for 3 h. Then 2 µg of primary antibody anti‐FLAG or anti‐PRMT5 was added into lysate contains 500 µg total protein and rotating at 4 °C overnight. The next morning added the protein A/G agarose and rotating for 3 h.

Total protein was extracted from HEK293T cells, transfected with pcDNA‐Myc‐Prm5 after 48 h, undifferentiated/differentiated 3T3‐L1 cell line. The lysate was precleared with protein A/G agarose at 4 °C for 3 h. Then 2 µg of primary antibody anti‐Spt5 was added into lysate contains 500 µg total protein and rotating at 4 °C overnight. The next morning added the protein A/G agarose and rotating for 3 h. The samples were washed with cold PBS for three times and collected for Western blot.

##### Immunoprecipitation and Silver Staining

Total protein was extracted from eWAT of Prmt5 KO and WT mice. Briefly, the 200 mg of fat tissue was collected from each mouse and homogenized with 1 mL lysis buffer (50 mm Tris‐HCl, pH 7.5, 150 mm NaCl, and 1% Nonidet P‐40 with 1 × protease inhibitor mixture) on ice. After another 10 min on ice, tissue lysate was centrifuged with 14 000 rpm at 4 °C, and the middle layer (containing 4 mg proteins determined by the Pierce bicinchoninic acid assay) was transferred to 50 µL BSA blocked Protein A/G agarose. After incubation on a rotator for 3 h in a cold room (4 °C), the beads were washed out and then 2 µg of anti‐Spt5 or anti‐SYM10 were added, anti‐mouse IgG or anti‐rabbit IgG were added as controls, respectively. After incubation on a rotator for overnight in a cold room (4 °C), 50 µL BSA blocked Protein A/G agarose was added and kept incubating for another 3 h. The beads were then washed using high and low salt lysis buffer for six times. Beads captured proteins were eluted with 50 µL of low salt lysis buffer and equal volume of 2× protein loading buffer on a heater (99 °C, 10 min). Proteins were separated by 12% SDS–PAGE. For silver staining, the gel was kept in a fixing solution (50% methanol, 12% acetic acid, and 0.05% formaldehyde in distilled water) under constant shaking at 4 °C overnight. Then the gel was washed for six times with ddH_2_O (5 min for each time). The gel was sensitized in 0.02% sodium thiosulfate solution for 2 min and then washed with ddH_2_O for three times (5 min for each time). The gel was kept in staining solution (0.1% silver nitrate and 0.076% formaldehyde) for 20 min in dark with rotation at room temperature. The gel was rinsed and then kept in a developing solution (6% Sodium Carbonate, 0.05% formaldehyde, and 0.0004% sodium thiosulfate) until the bands were visible. Stopping solution (50% methanol and 12% acetic acid) was poured on the gel to stop the reaction. The gel was imaged using a FluorChem R System (ProteinSimple).

##### Statistical Analysis

Trial experiments or experiments done previously were used to determine sample size with adequate statistical power. The researchers involved in the in vivo treatments were not completely blinded, but all ITT and GTT were conducted blindly. All images were randomly captured from sample and analyzed in a blinded manner. No data were excluded from statistical analysis. All experimental data are represented as mean ± s.e.m (*n *≥ 3) and statistical comparisons were based on Student's *t*‐test with a two‐tail distribution. Comparisons with *p* values <0.05 or <0.01 were considered statistically significant. Graphs were generated by GraphPad prism software.

## Conflict of Interest

The authors declare no conflict of interest.

## Author contributions

Z.J. and F.Y. contributed equally to this work. S.K. conceived the project. Z.J., F.Y., and S.K. designed the experiments and prepared the manuscript. Z.J., F.Y., X.C., N.N., J.Q., and S.S. performed the experiments and analyzed the data. P. Y. performed data mining and gene expression analysis. A.I., M.D., and C.H. provided key reagents and all authors revised the manuscript.

## Supporting information

Supporting Information
