# Supplementary material for: Protein Arginine Methyltransferase PRMT5 Regulates Fatty Acid Metabolism and Lipid Droplet Biogenesis in White Adipose Tissues
Source: Adv Sci (Weinh). 2020 Oct 16;7(23):2002602. doi: 10.1002/advs.202002602 (PMC7709973; doi:10.1002/advs.202002602)
Supplement: Supplementary file 1 — Supporting Information [file ADVS-7-2002602-s001.pdf]

© 2020 Wiley-VCH GmbH

ADVANCED SCIENCE Open Access

Supporting Information

for *Adv. Sci.,* DOI: 10.1002/advs.202002602

Protein arginine methyltransferase PRMT5 regulates fatty acid metabolism and lipid droplet biogenesis in white adipose tissues

*Dr. Zhihao Jia, Dr. Feng Yue, Xiyue Chen, Dr. Naagarajan Narayanan, Jiamin Qiu, Dr. Sabriya A. Syed, Prof. Anthony N. Imbalzano, Prof. Meng Deng, Prof. Peng Yu, Prof. Changdeng Hu, and Prof. Shihuan Kuang**

Supplemental information

Protein arginine methyltransferase PRMT5 regulates fatty acid metabolism and lipid droplet biogenesis in white adipose tissues

*Dr. Zhihao Jia[1, #], Dr. Feng Yue[1, #], Xiyue Chen[1], Dr. Naagarajan Narayanan[2,3], Jiamin Qiu[1], Dr. Sabriya A. Syed[4], Prof. Anthony N. Imbalzano[4], Prof. Meng Deng[2,3], Prof. Peng Yu[5,6], Prof. Changdeng Hu[7,8], and Prof. Shihuan Kuang[1,8, *]*

[1] Department of Animal Sciences, Purdue University, West Lafayette, Indiana, 47907, USA.
[2] Department of Agricultural and Biological Engineering, [3]Bindley Bioscience Center, Purdue University, West Lafayette, Indiana, 47907, USA.
[4] Department of Biochemistry and Molecular Pharmacology, University of Massachusetts Medical School, Worcester, Massachusetts, USA.
[5] West China Biomedical Big Data Center, West China Hospital, Sichuan University, Chengdu, China
[6] Medical Big Data Center, Sichuan University, Chengdu, China.
[7] Department of Medicinal Chemistry and Molecular Pharmacology, Purdue University, West Lafayette, Indiana, 47907, USA.
[8] Purdue University Center for Cancer Research, West Lafayette, Indiana, 47907, USA.
[#]Equal contribution
*Correspondence

The following files are included:

Supplemental Figures (Figure S1-S11) and Figure Legends

Supplemental Tables (Table S1-S2)

Supplemental figures and figure legends

Figure S1

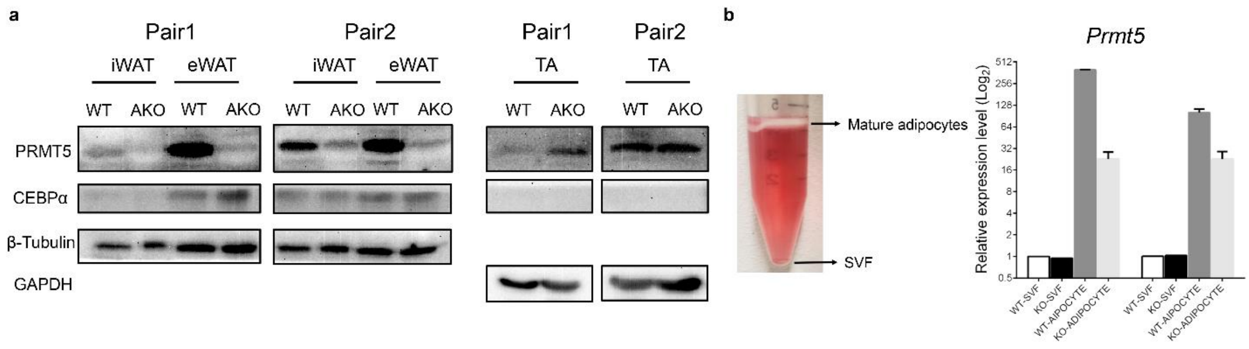

Figure S1. Specific knockout of Prmt5 in Prmt5^{AKO} mice.

(a) Representative western blots showing protein levels of PRMT5, CEBPα, GAPDH and β-Tubulin from iWAT, eWAT and TA muscle of 3-month-old male WT and Prmt5^{AKO} mice. (b) Expression levels of *Prmt5* in SVF preadipocytes and mature adipocytes from iWAT and eWAT of 8-week-old male WT and Prmt5^{AKO} mice. Data represent mean ± s.e.m.

Figure S2

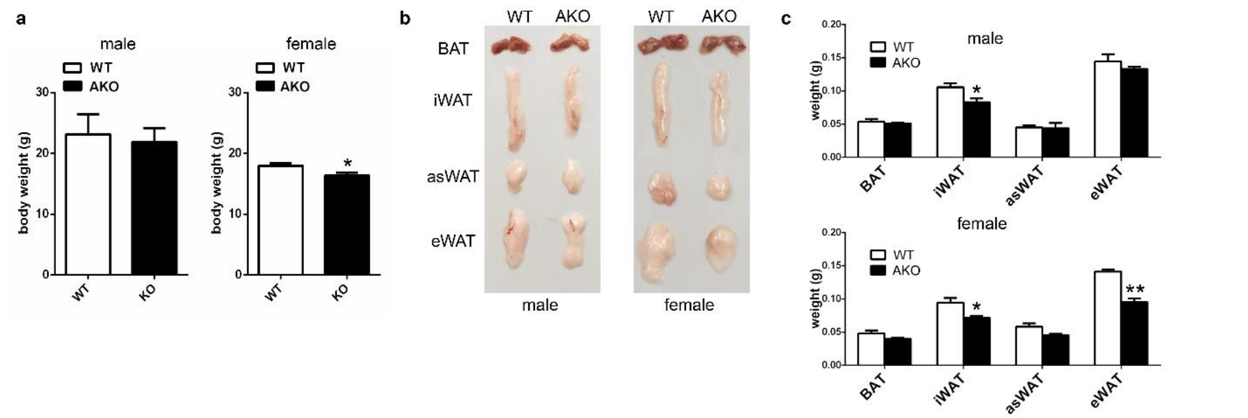

Figure S2. Prmt5^{AKO} mice have less fat at 8-week old.

(a) Reduction of body weight of female Prmt5^{AKO} mice relative to WT mice (right panel), n=4 pairs of mice for 8-week-old. (b) Representative images of BAT and WAT depots showing mass reduction of Prmt5 KO WAT at 8-week-old. (c) Weights of various BAT and WAT depots at 8-week-old. Data represent mean ± s.e.m. (t-test: * P<0.05, **P<0.01).

Figure S3

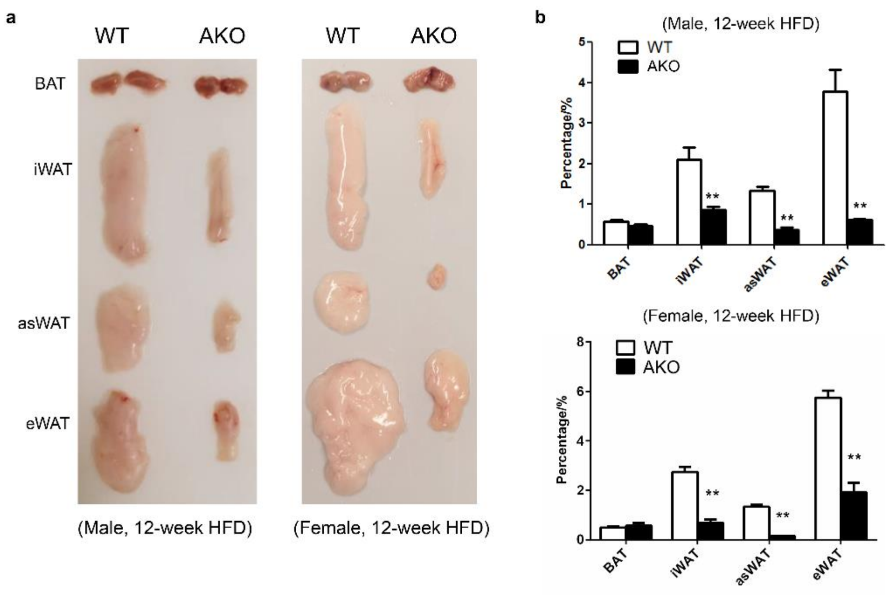

Figure S3. Specific reduction of *Prmt5* KO WAT after 12-week HFD.

(a) Representative images of BAT and WAT depots showing mass reduction of WAT in both male and female Prmt5^{AKO} mice after 12-week HFD. (b) WAT and BAT relative weights normalized to body weight from male (upper panel) and female (lower panel) WT (Prmt5$^{flox/flox}$) and Prmt5^{AKO} mice after 12-week of HFD, n=5 pairs of mice. Data represent mean ± s.e.m. (t-test: **P<0.01).

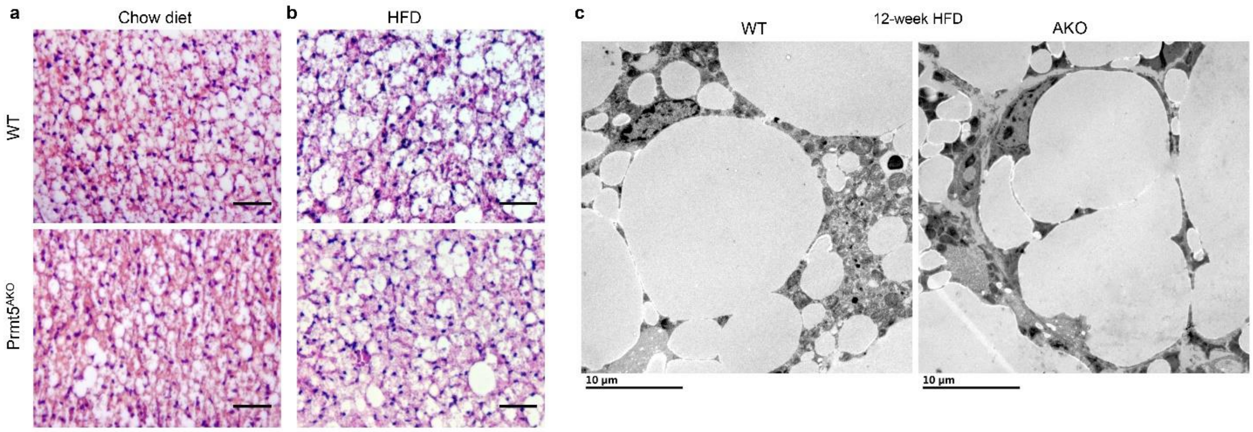

Figure S4. Prmt5^{AKO} has minor effect on LD size of BAT.

(a, b) H&E staining of BAT sections from male WT (Prmt5$^{flox/flox}$) and Prmt5^{AKO} mice at 6-month-old (a) or after 12-week of HFD (b), respectively. Scale bar: 50 μm. (c) Representative Transmission Electron Microscopy (TEM) images of BAT from 12-week HFD fed male WT (Prmt5$^{flox/flox}$) and Prmt5^{AKO} mice.

Figure S5

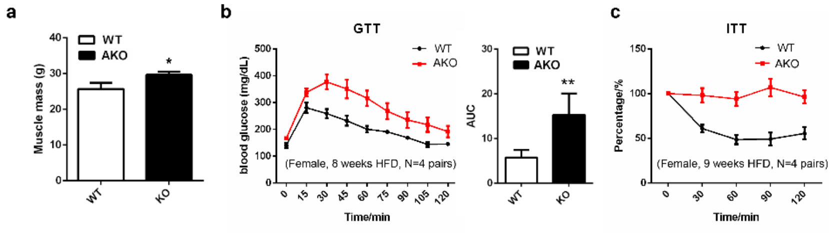

Figure S5. Prmt5^{AKO} causes defects in glucose and insulin sensitivity.

(a) Compared to WT mice, Prmt5^{AKO} leads to increase of total body lean mass at 10-month-old, n=5 pairs. (b) Blood glucose concentrations during glucose tolerance tests (GTT) performed on WT (Prmt5$^{flox/flox}$) and Prmt5^{AKO} mice after 8-week of HFD (left). Area under curve (AUC) calculated based on data in left panel (right), n=4 pairs of female mice. (c) Percentage changes of blood glucose concentrations during insulin tolerance tests (ITT) performed on WT (Prmt5$^{flox/flox}$) and Prmt5^{AKO} mice after 9-week of HFD, n=5 pairs of female mice. Data represent mean ± s.e.m. (t-test: **P<0.05).

Figure S6

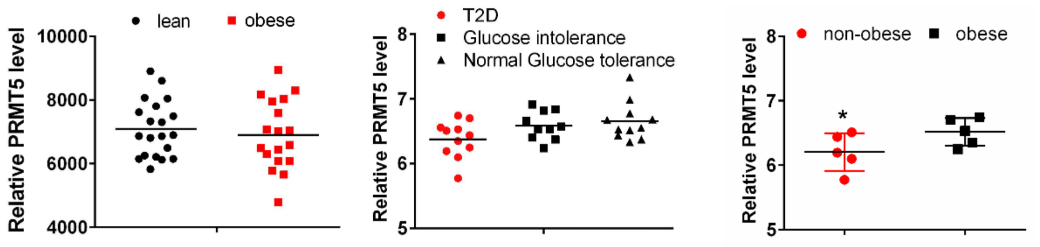

Figure S6. *PRMT5* expression is corelated to non-obese T2D patients.

(a) *PRMT5* expression levels in WAT from normal lean and obese people. (b) *PRMT5* expression levels in WAT from normal glucose tolerance and glucose intolerance people, and T2D patients. (c) *PRMT5* expression levels in WAT from non-obese and obese T2D patients. Data represent mean ± s.e.m. (t-test: * P<0.05).

Figure S7

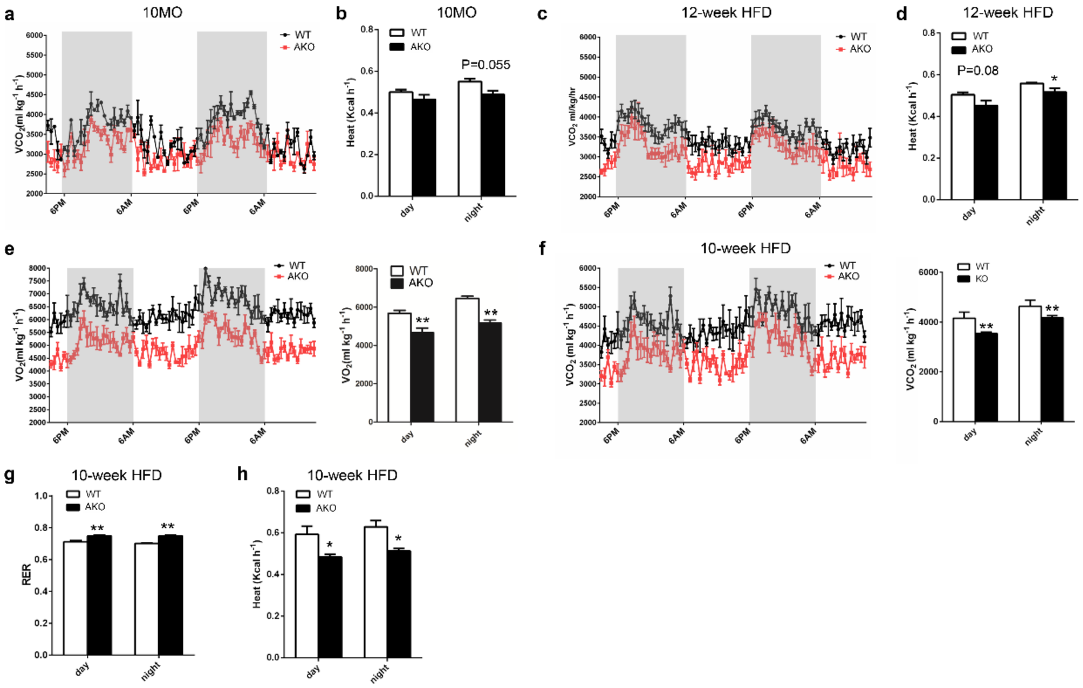

Figure S7. Prmt5^{AKO} causes systemic metabolic defect.

(a) CO2 production measured by an indirect calorimetry and normalized to lean mass is shown for a 48-hour cycle of 10-month-old male WT (Prmt5$^{flox/flox}$) and Prmt5^{AKO} mice. (b) Average day and night heat production, n=5 pairs of 10-month-old male mice. (c) CO2 production measured same as above on WT (Prmt5$^{flox/flox}$) and Prmt5^{AKO} mice after 12-week of HFD. (d) Average day and night heat production, n=6 pairs of male mice after 12-week of HFD. (e-h) O2 consumption (e), CO2 production (f), RER (g) and heat production (h) measured same as above on female WT (Prmt5$^{flox/flox}$) and Prmt5^{AKO} mice after 10-week of HFD. n=4 pairs of female mice after 10-week of HFD. Data represent mean ± s.e.m. (t-test: *P<0.05, **P<0.01).

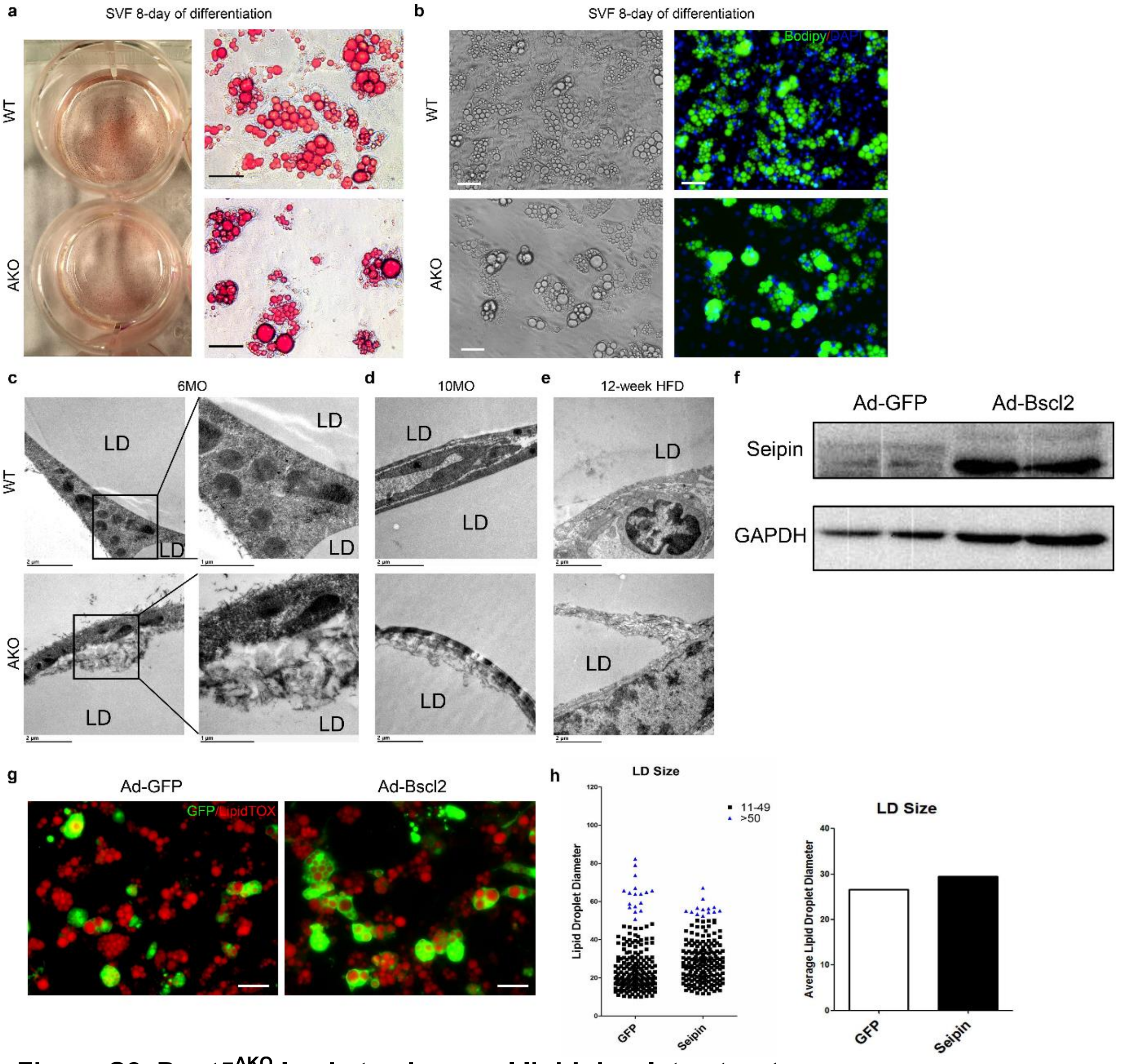

Figure S8. Prmt5^{AKO} leads to abnormal lipid droplets structure.

(a) Oil red staining of SVF preadipocytes from eWAT of 8-week-old male WT (Prmt5$^{flox/flox}$) and Prmt5^{AKO} mice after 8-day differentiation. Scale bar: 50 µm. (b) Representative phase contrast (left) and Bodipy and DAPI staining (right) images of 8-day differentiated SVF preadipocytes from eWAT of 8-week-old male WT (Prmt5$^{flox/flox}$) and Prmt5^{AKO} mice. Scale bar: 50 µm. (c-e) Representative TEM images of eWAT from mice WT (Prmt5$^{flox/flox}$) and Prmt5^{AKO} mice at 6- (c) and 10-month-old (d), 12-week HFD fed (e). (f) WB showing the overexpression efficiency of Bscl2 Adenovirus. (g) Representative images of SVF preadipocytes from WT mice after 10-day of differentiation treated with GFP-expressing adenovirus (control) or Bscl2/GFP expressing adenovirus, scale bar: 50 µm. (h) Size distribution and average diameters of lipid droplets in GFP adenovirus or Bscl2/GFP adenovirus treated SVF preadipocytes from WT mice after 10-day of differentiation.

Figure S9

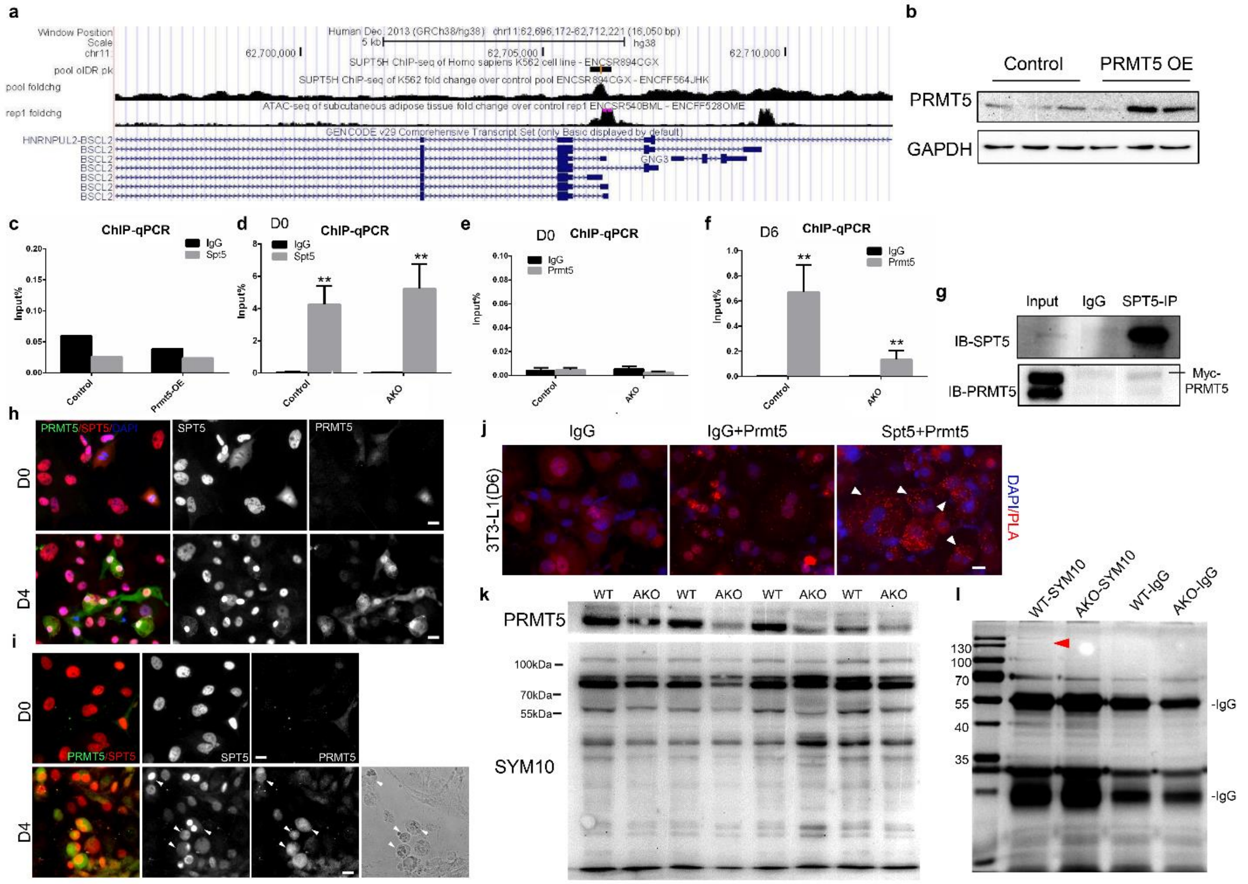

Figure S9. PRMT5 interacts with and methylates the transcriptional pausing factor SPT5 to affect its binding to the *Bscl2* TSS.

(a) SPT5 ChIP-seq and ATAC-seq showing a SPT5 binding at human *BSCL2* TSS. (b) Overexpression efficiency of PRMT5. (c) SPT5 ChIP-qPCR of MG53 promoter region. (d) ChIP-qPCR analysis of SPT5 binding to the *Bscl2* TSS region in undifferentiated (D0) SVF preadipocytes from 8-week-old male WT ($Prmt5^{flox/flox}$) and $Prmt5^{AKO}$ mice. (e, f) ChIP-qPCR analysis of PRMT5 binding to the *Bscl2* TSS region in undifferentiated (D0) (e) and differentiated (D6) (f) SVF preadipocytes from WT ($Prmt5^{flox/flox}$) and $Prmt5^{AKO}$ mice. (g) Lysates from PRMT5 overexpressed 293T cells were immunoprecipitated (IP) with SPT5 antibody and bolted using the SPT5 and PRMT5 antibodies. (h, i) Co-staining of SPT5 and PRMT5 in undifferentiated (D0) and differentiated (D4) 3T3-L1 cells (h) and SVF preadipocytes (i). White arrowheads indicates the differentiated 3T3-L1 cells, scale bar: 20 µm. (j) Proximity ligation assay (PLA) using PRMT5 and SPT5 antibodies in differentiated 3T3-L1 cells, scale bar: 20 µm. (k) WB using SYM10 and PRMT5 antibodies of protein lysates from WT ($Prmt5^{flox/flox}$) and $Prmt5^{AKO}$ mice. (l) Sliver staining of proteins that were immunoprecipitated (IP) by SYM10 antibody from eWAT of male WT ($Prmt5^{flox/flox}$) and $Prmt5^{AKO}$ mice at 2-month-old. Red arrow shows a potential SPT5 band. Data represent mean ± s.e.m. (t-test: **P<0.01).

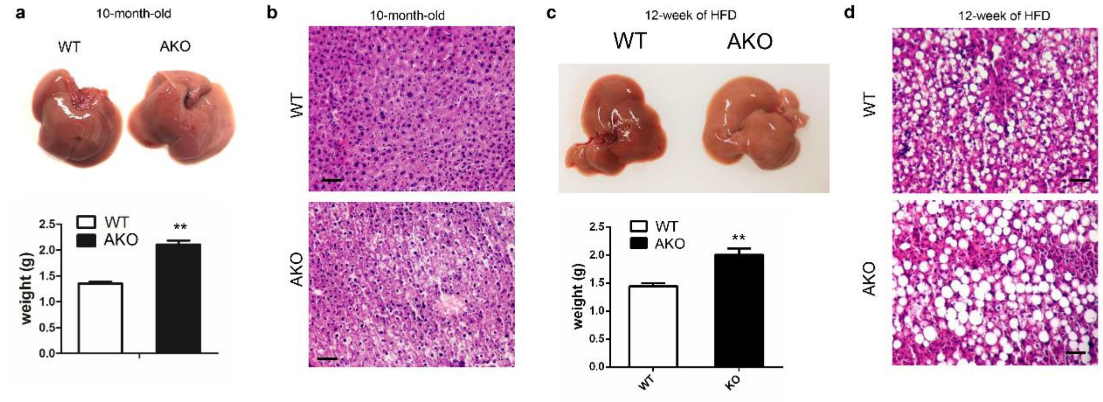

Figure S10. Characterization of hepatic steatosis in Prmt5^{AKO} mice.

(a) Representative images of liver from 10-month-old male WT (Prmt5$^{flox/flox}$) and Prmt5^{AKO} mice (upper), increase of liver weight of Prmt5^{AKO} mice relative to WT mice (lower). n=3 pairs of male mice. (b) H&E staining of liver sections from 10-month-old male WT (Prmt5$^{flox/flox}$) and Prmt5^{AKO} mice, scale bar: 50 μm. (c) Representative images of liver from WT (Prmt5$^{flox/flox}$) and Prmt5^{AKO} mice (upper), increase of liver weight of Prmt5^{AKO} mice relative to WT mice (lower) after 12-week of HFD. n=6 pairs of male mice. (d) H&E staining of liver sections from male WT (Prmt5$^{flox/flox}$) and Prmt5^{AKO} mice after 12-week of HFD, scale bar: 50 μm. Data represent mean ± s.e.m. (t-test: **P<0.01).

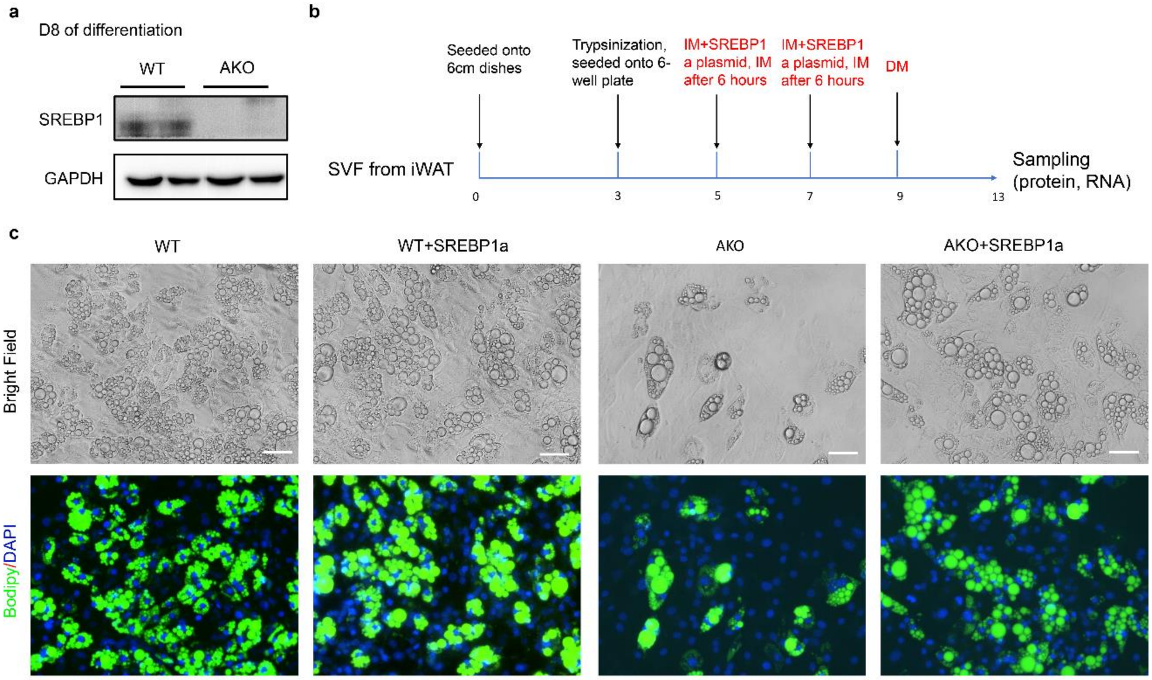

Figure S11. PRMT5 regulates the lipid synthesis through SREBP1.

(a) Representative western blots showing the protein level of SREBP1 and GAPDH in SVF preadipocytes from iWAT of 8-week-old male WT (Prmt5$^{flox/flox}$) and Prmt5^{AKO} mice after 8-day of differentiation in vitro.(b) Overexpression strategy of Flag-SREBP1a in SVF preadipocytes during differentiation. (c) Representative phase contrast (upper) and Bodipy and DAPI staining (lower) images of 8-day differentiated SVF preadipocytes from eWAT of 8-week-old male WT (Prmt5$^{flox/flox}$) and Prmt5^{AKO} mice with overexpression of pcDNA3.1-Flag or Flag-SREBP1a, Scale bar: 50 μm.

Supplemental Tables

Table S1. Lipidomics identification of triacylglycerides (TAG) and their relative levels (KO/WT) in *Prmt5* KO white adipose tissues compared to wildtype (WT) control tissues.

**Table S1. TAG from 6-month-old mice**		
**Name**	**Fold Change**	**log2(FC)**
TAG482_FA 161	0.32003	-1.6437
TAG483_FA 161	0.36913	-1.4378
TAG481_FA 161	0.48345	-1.0486
TAG504_FA 161	0.4851	-1.0437
TAG523_FA 161	0.49506	-1.0143
TAG503_FA 161	0.50446	-0.98719
TAG524_FA 161	0.50774	-0.97784
TAG482_FA 181	0.52121	-0.94007
TAG541_FA 180	1.8613	0.89634
TAG547_FA 161	0.54295	-0.88111
TAG526_FA 161	0.56107	-0.83375
TAG481_FA 160	0.5672	-0.81808
TAG567_FA 161	0.56865	-0.8144
TAG502_FA 161	0.57062	-0.8094
TAG525_FA 161	0.57264	-0.8043
TAG503_FA 181	0.57824	-0.79026
TAG546_FA 161	0.58181	-0.78138
TAG525_FA 181	0.58666	-0.7694
TAG545_FA 161	0.60722	-0.71971
TAG563_FA 180	1.6412	0.71476
TAG524_FA 181	0.61082	-0.71118
TAG566_FA 161	0.61336	-0.70519
TAG548_FA 161	0.61488	-0.70162
TAG568_FA 161	0.6195	-0.69083
TAG504_FA 160	0.61969	-0.69038
TAG520_FA 180	1.6073	0.68466
TAG544_FA 180	1.5945	0.67313
TAG483_FA 160	0.63162	-0.66287
TAG541_FA 181	1.5825	0.66221
TAG520_FA 160	1.5625	0.64387
TAG521_FA 180	1.5218	0.60582
TAG542_FA 180	1.5113	0.59583
TAG480_FA 161	0.66377	-0.59125
TAG522_FA 161	0.66851	-0.58098

**Table S1. TAG from HFD mice**		
**Name**	**Fold Change**	**log2(FC)**
TAG483_FA 181	0.55166	-0.85816
TAG526_FA 161	0.57232	-0.80509
TAG483_FA 161	0.57841	-0.78983
TAG483_FA 160	0.59337	-0.753
TAG482_FA 161	0.61614	-0.69866
TAG482_FA 181	0.62786	-0.67148
TAG587_FA 160	0.63538	-0.65431
TAG547_FA 160	0.64144	-0.64062
TAG481_FA 161	0.6435	-0.63599
TAG541_FA 161	0.64875	-0.62428
TAG567_FA 161	0.6532	-0.6144
TAG546_FA 161	0.65852	-0.6027
TAG565_FA 160	0.65932	-0.60094
TAG567_FA 160	0.65944	-0.60068
TAG604_FA 160	0.66132	-0.59659
TAG602_FA 180	0.66259	-0.59381
TAG561_FA 160	0.66326	-0.59235
TAG568_FA 160	0.66461	-0.58943
TAG568_FA 180	0.66566	-0.58714
TAG544_FA 160	0.66584	-0.58675
TAG504_FA 181	0.6672	-0.5838
TAG581_FA 180	0.6683	-0.58142
TAG583_FA 180	0.66878	-0.58039
TAG482_FA 160	0.66943	-0.579
TAG567_FA 180	0.67017	-0.57739
TAG6010_FA 180	0.67071	-0.57623
TAG606_FA 160	0.67075	-0.57615
TAG504_FA 180	0.67109	-0.57542
TAG608_FA 160	0.67115	-0.5753
TAG607_FA 160	0.67274	-0.57189
TAG582_FA 161	0.67471	-0.56766
TAG562_FA 161	0.67586	-0.5652
TAG609_FA 160	0.67605	-0.56479
TAG584_FA 180	0.67728	-0.56218

TAG526_FA 181	0.66891	-0.58011
TAG541_FA 161	0.67381	-0.56959
TAG504_FA 181	0.67507	-0.56689
TAG521_FA 160	1.4772	0.5629
TAG522_FA 180	1.4729	0.55862
TAG543_FA 180	1.4722	0.55796
TAG604_FA 160	0.68166	-0.55288
TAG589_FA 161	0.68223	-0.55168
TAG483_FA 181	0.68733	-0.54092
TAG542_FA 161	0.68923	-0.53694
TAG584_FA 161	0.69253	-0.53006
TAG587_FA 161	0.69846	-0.51776
TAG563_FA 161	0.70016	-0.51424
TAG524_FA 180	0.70219	-0.51007
TAG562_FA 180	1.4229	0.50882
TAG609_FA 161	0.7046	-0.50512
TAG564_FA 180	1.4186	0.50443
TAG560_FA 161	0.71448	-0.48504
TAG562_FA 161	0.71508	-0.48383
TAG481_FA180	0.71566	-0.48265
TAG540_FA 161	0.72174	-0.47046
TAG604_FA 161	0.72202	-0.46988
TAG604_FA 180	0.72286	-0.4682
TAG526_FA 180	0.72322	-0.4675
TAG544_FA 161	0.72373	-0.46647
TAG543_FA 160	1.3786	0.4632
TAG582_FA 161	0.72707	-0.45984
TAG565_FA 161	0.72748	-0.45902
TAG545_FA 180	1.3745	0.45893
TAG541_FA 160	1.3736	0.458
TAG605_FA 161	0.72882	-0.45636
TAG548_FA 180	1.3704	0.45457
TAG603_FA 161	0.73137	-0.45133
TAG520_FA 181	1.3664	0.45037
TAG584_FA 160	0.73367	-0.44679
TAG500_FA 160	1.3606	0.44421
TAG601_FA 161	0.7352	-0.4438
TAG600_FA 160	0.73628	-0.44167
TAG6011_FA 161	0.7373	-0.43967
TAG600_FA 180	0.73862	-0.43709
TAG6012_FA 180	0.73893	-0.43649
TAG504_FA 180	0.74277	-0.42902
TAG500_FA 161	0.74321	-0.42816

TAG548_FA 160	0.67823	-0.56015
TAG568_FA 161	0.67886	-0.55882
TAG526_FA 180	0.67903	-0.55846
TAG588_FA 180	0.67924	-0.55801
TAG548_FA 161	0.6797	-0.55702
TAG561_FA 161	0.68038	-0.55558
TAG566_FA 161	0.68053	-0.55526
TAG587_FA 161	0.68084	-0.55462
TAG581_FA 161	0.68095	-0.55439
TAG525_FA 180	0.68104	-0.5542
TAG580_FA 160	0.68195	-0.55225
TAG601_FA 180	0.68247	-0.55116
TAG6011_FA 160	0.68295	-0.55015
TAG603_FA 161	0.68301	-0.55002
TAG589_FA 160	0.68354	-0.5489
TAG600_FA 160	0.68357	-0.54884
TAG6011_FA 161	0.68358	-0.54882
TAG585_FA 161	0.68419	-0.54753
TAG483_FA 180	0.68429	-0.54731
TAG6010_FA 161	0.68455	-0.54678
TAG546_FA 181	0.68463	-0.5466
TAG589_FA 180	0.68468	-0.54651
TAG605_FA 180	0.6849	-0.54603
TAG587_FA 180	0.68517	-0.54546
TAG606_FA 180	0.68546	-0.54485
TAG586_FA 161	0.68598	-0.54377
TAG577_FA 161	0.68614	-0.54342
TAG600_FA 180	0.68634	-0.543
TAG606_FA 161	0.68644	-0.54279
TAG5810_FA 161	0.68697	-0.54168
TAG584_FA 160	0.68733	-0.54092
TAG609_FA 180	0.68746	-0.54066
TAG547_FA 161	0.68752	-0.54052
TAG480_FA 180	0.68761	-0.54034
TAG6012_FA 180	0.68777	-0.54
TAG597_FA 161	0.68793	-0.53967
TAG602_FA 161	0.68835	-0.53878
TAG6012_FA 161	0.68899	-0.53744
TAG6010_FA 160	0.68904	-0.53733
TAG607_FA 161	0.6893	-0.5368
TAG617_FA 161	0.6893	-0.5368
TAG560_FA 160	0.68944	-0.5365
TAG589_FA 161	0.68955	-0.53626

TAG607_FA 161	0.74466	-0.42536
TAG586_FA 161	0.74535	-0.42401
TAG5810_FA 161	0.7482	-0.41851
TAG521_FA 181	1.3363	0.41823
TAG588_FA 161	0.74837	-0.41817
TAG501_FA 180	1.3343	0.4161
TAG580_FA 161	0.7503	-0.41446
TAG586_FA 160	0.75121	-0.41271
STD_150-181d7-150 TAG	0.75152	-0.41211
TAG601_FA 160	0.75283	-0.40961
TAG521_FA 161	0.75324	-0.40882
TAG520_FA 161	0.75554	-0.40443
TAG6012_FA 161	0.75887	-0.39808
TAG542_FA 181	1.3173	0.39759
TAG543_FA 161	0.76009	-0.39575
TAG6011_FA 180	0.7602	-0.39556
TAG6010_FA 180	0.7618	-0.39252
TAG525_FA 180	0.76263	-0.39094
TAG542_FA 160	1.3085	0.38794
TAG561_FA 161	0.76858	-0.37973
TAG606_FA 160	0.76868	-0.37954
TAG564_FA 161	0.76954	-0.37794
TAG603_FA 180	0.77012	-0.37684
TAG581_FA 161	0.77156	-0.37415
TAG482_FA 160	0.77233	-0.37271
TAG605_FA 160	0.77414	-0.36934
TAG603_FA 160	0.77651	-0.36492
TAG607_FA 160	0.77777	-0.36258
TAG6010_FA 161	0.77933	-0.3597
TAG6012_FA 160	0.78012	-0.35823
TAG585_FA 161	0.78049	-0.35755
TAG609_FA 180	0.78304	-0.35283
TAG6011_FA 160	0.7843	-0.35052
TAG608_FA 161	0.78593	-0.34753
TAG600_FA 161	0.78669	-0.34612
TAG606_FA 180	0.78804	-0.34367
TAG585_FA 160	0.7885	-0.34282
TAG583_FA 161	0.78876	-0.34234
TAG483_FA 180	0.78955	-0.34089
TAG6010_FA 160	0.79031	-0.33952
TAG582_FA 180	0.79257	-0.3354
TAG608_FA 180	0.79326	-0.33413

TAG5810_FA 160	0.68971	-0.53593
TAG6011_FA 180	0.68993	-0.53548
TAG557_FA 161	0.68995	-0.53544
TAG608_FA 180	0.69	-0.53534
TAG566_FA 160	0.69018	-0.53496
TAG583_FA 161	0.69052	-0.53425
TAG603_FA 160	0.69074	-0.53379
TAG537_FA 161	0.69145	-0.5323
TAG564_FA 161	0.69239	-0.53033
TAG605_FA 160	0.69305	-0.52897
TAG565_FA 161	0.69311	-0.52884
TAG605_FA 161	0.69322	-0.5286
TAG582_FA 160	0.69357	-0.5279
TAG581_FA 160	0.695	-0.52491
TAG601_FA 160	0.69503	-0.52486
TAG607_FA 180	0.69515	-0.5246
TAG6012_FA 160	0.69568	-0.5235
TAG517_FA 161	0.69772	-0.51928
TAG601_FA 161	0.69879	-0.51707
TAG604_FA 161	0.69891	-0.51683
TAG546_FA 180	0.69941	-0.51578
TAG582_FA 180	0.69951	-0.51559
TAG609_FA 161	0.69976	-0.51506
TAG608_FA 161	0.70015	-0.51426
TAG580_FA 180	0.70112	-0.51227
TAG547_FA 181	0.70118	-0.51215
TAG5810_FA 180	0.70157	-0.51134
TAG585_FA 180	0.70236	-0.50973
TAG545_FA 180	0.70258	-0.50928
TAG588_FA 161	0.70304	-0.50832
TAG521_FA 161	0.70461	-0.5051
TAG526_FA 160	0.70533	-0.50364
TAG584_FA 161	0.70586	-0.50255
TAG481_FA180	0.70587	-0.50253
TAG563_FA 161	0.70707	-0.50007
TAG602_FA 160	0.70804	-0.49811
TAG583_FA 160	0.70831	-0.49754
TAG603_FA 180	0.70834	-0.49748
TAG560_FA 180	0.71108	-0.49191
TAG586_FA 160	0.71142	-0.49123
TAG588_FA 160	0.71304	-0.48795
TAG504_FA 161	0.71352	-0.48698

TAG480_FA 181	0.79712	-0.32713
TAG525_FA 160	1.2434	0.3143
TAG609_FA 160	0.80447	-0.31389
TAG580_FA 180	0.80534	-0.31233
TAG580_FA 160	0.80684	-0.30965
TAG602_FA 180	0.80805	-0.30748
TAG608_FA 160	0.80831	-0.30702
TAG567_FA 180	0.81411	-0.29671
TAG568_FA 160	0.8146	-0.29584
TAG548_FA 181	1.2242	0.29189
TAG607_FA 180	0.81708	-0.29145
TAG602_FA 160	0.83135	-0.26648
TAG522_FA 160	1.1988	0.26154
TAG5810_FA 180	0.83442	-0.26116
TAG524_FA 160	1.1953	0.25742
TAG544_FA 160	1.1935	0.25517
TAG547_FA 160	0.84247	-0.2473
TAG560_FA 160	0.84491	-0.24314
TAG502_FA 180	0.84676	-0.23998
TAG546_FA 180	0.84901	-0.23614
TAG481_FA 181	0.85083	-0.23307
TAG586_FA 180	1.171	0.22772
TAG581_FA 160	0.86074	-0.21635
TAG583_FA 180	0.87029	-0.20043
TAG523_FA 180	0.87051	-0.20006
TAG587_FA 180	0.87219	-0.19728
TAG581_FA 180	0.87757	-0.18841
TAG482_F A180	0.87789	-0.18789
TAG566_FA 180	1.1312	0.17787
TAG501_FA 181	1.1244	0.1691
TAG588_FA 160	0.89039	-0.16749
TAG588_FA 180	0.89117	-0.16623
TAG523_FA 160	1.1211	0.16491
TAG547_FA 180	0.89385	-0.1619
TAG561_FA 160	1.1156	0.15782
TAG503_FA 160	1.1152	0.15731
TAG567_FA 160	0.89853	-0.15436
TAG5810_FA 160	0.89917	-0.15334
TAG587_FA 160	0.9028	-0.14751
TAG560_FA 180	0.90389	-0.14578
TAG589_FA 160	0.90656	-0.14152
TAG502_FA 160	1.0978	0.13467
TAG543_FA 181	0.91098	-0.13451

TAG547_FA 180	0.71397	-0.48607
TAG526_FA 181	0.71416	-0.48569
TAG525_FA 160	0.71551	-0.48296
TAG546_FA 160	0.71692	-0.48012
TAG545_FA 161	0.72183	-0.47028
TAG543_FA 161	0.72215	-0.46964
TAG585_FA 160	0.72232	-0.46929
TAG566_FA 180	0.7224	-0.46913
TAG481_FA 160	0.72245	-0.46903
TAG586_FA 180	0.72484	-0.46426
TAG497_FA 181	0.72746	-0.45907
TAG503_FA 180	0.72773	-0.45852
TAG564_FA 160	0.72797	-0.45805
TAG604_FA 180	0.72963	-0.45477
TAG540_FA 160	0.73044	-0.45316
TAG504_FA 160	0.73124	-0.45157
TAG545_FA 160	0.73222	-0.44965
TAG525_FA 161	0.73308	-0.44795
TAG480_FA 160	0.73959	-0.4352
TAG565_FA 180	0.74002	-0.43437
TAG481_FA 181	0.74436	-0.42593
TAG563_FA 160	0.74841	-0.41809
TAG482_F A180	0.75176	-0.41166
TAG544_FA 161	0.75202	-0.41116
TAG524_FA 180	0.75561	-0.40429
TAG542_FA 161	0.75707	-0.4015
TAG562_FA 160	0.75936	-0.39714
TAG541_FA 160	0.76059	-0.39481
TAG524_FA 181	0.76676	-0.38315
TAG503_FA 181	0.77236	-0.37266
TAG503_FA 161	0.77459	-0.36849
TAG557_FA 181	0.78016	-0.35816
TAG525_FA 181	0.78251	-0.35382
TAG524_FA 161	0.79422	-0.33239
TAG502_FA 180	0.8108	-0.30258
TAG502_FA 161	0.81225	-0.3
TAG523_FA 180	0.81653	-0.29243
TAG561_FA 180	0.81997	-0.28635
TAG541_FA 181	1.1982	0.26088
TAG564_FA 180	0.83946	-0.25247
TAG542_FA 181	1.1874	0.24781
TAG542_FA 180	1.1851	0.24501
TAG543_FA 160	0.84402	-0.24466

TAG589_FA 180	0.91281	-0.13161
TAG584_FA 180	0.91454	-0.12888
TAG565_FA 160	1.0914	0.12623
TAG568_FA 180	1.0912	0.12591
TAG585_FA 180	0.91669	-0.12549
TAG565_FA 180	1.0892	0.12324
TAG544_FA 181	0.92042	-0.11964
TAG561_FA 180	1.0766	0.10654
TAG502_FA 181	1.0755	0.10498
TAG583_FA 160	1.0721	0.10048
TAG546_FA 181	1.069	0.096234
TAG540_FA 160	0.93548	-0.096216
TAG480_FA 160	1.0627	0.087773
TAG563_FA 160	1.0626	0.087566
TAG547_FA 181	0.94149	-0.086977
TAG526_FA 160	1.0567	0.079546
TAG562_FA 160	1.0515	0.072508
TAG540_FA 181	1.0464	0.065448
TAG522_FA 181	1.0436	0.061521
TAG545_FA 160	1.0404	0.057121
TAG548_FA 160	1.0374	0.05293
TAG566_FA 160	1.0371	0.052561
TAG546_FA 160	0.96473	-0.051799
TAG523_FA 181	0.96682	-0.048675
TAG545_FA 181	0.96953	-0.044648
TAG564_FA 160	1.0304	0.043198
TAG480_FA 180	1.0231	0.032918
TAG582_FA 160	1.0133	0.019063
TAG503_FA 180	0.98965	-0.015004

TAG541_FA 180	1.1811	0.24011
TAG523_FA 161	0.85155	-0.23183
TAG497_FA 161	0.86346	-0.2118
TAG563_FA 180	0.87178	-0.19796
TAG501_FA 180	0.87387	-0.19451
TAG522_FA 161	0.87866	-0.18662
TAG545_FA 181	0.89932	-0.1531
TAG524_FA 160	0.90498	-0.14404
TAG501_FA 181	0.9058	-0.14273
TAG540_FA 180	0.90598	-0.14245
TAG521_FA 160	1.1032	0.14175
TAG521_FA 180	1.098	0.13482
TAG543_FA 181	1.0958	0.13199
TAG548_FA 181	0.91541	-0.12751
TAG521_FA 181	1.0896	0.12373
TAG522_FA 160	1.0891	0.12317
TAG543_FA 180	1.0878	0.12146
TAG544_FA 180	0.92688	-0.10955
TAG503_FA 160	0.92705	-0.10928
TAG522_FA 181	1.0776	0.10779
TAG542_FA 160	0.92929	-0.10579
TAG537_FA 181	0.94308	-0.084554
TAG520_FA 160	1.0493	0.069404
TAG523_FA 181	0.95452	-0.067145
TAG548_FA 180	0.96018	-0.058619
TAG500_FA 160	0.96323	-0.054043
TAG502_FA 181	0.96951	-0.044675
TAG523_FA 160	1.0236	0.033698
TAG562_FA 180	1.0196	0.028025
TAG522_FA 180	1.0064	0.0092598
TAG544_FA 181	1.0063	0.009056
TAG502_FA 160	1.0044	0.0062791
TAG520_FA 180	0.999	-0.0014386

Table S2. Primers used in this study.

Primer	Sequence (5'—3')
**Genotyping PCR**	
Prmt5	F: ACTGGGTTGCTCACAACTGC
	R: GGGAGCCCACCTTACCTG
**Real-time PCR**	
qPrmt5	F: CTGAATTGCGTCCCCGAAATA
	R: AGGTTCCTGAATGAACTCCCT
qUcp1	F: AGGCTTCCAGTACCATTAGGT
	R: CTGAGTGAGGCAAAGCTGATTT
qPparγ	F: TCGCTGATGCACTGCCTATG
	R: GAGAGGTCCACAGAGCTGATT
qC/EBPα	F: CAAGAACAGCAACGAGTACCG
	R: GTCACTGGTCAACTCCAGCAC
qFasn	F: GGAGGTGGTGATAGCCGGTAT
	R: TGGGTAATCCATAGAGCCCAG
qDgat1	F: TCCGTCCAGGGTGGTAGTG
	R: TGAACAAAGAATCTTGCAGACGA
qDgat2	F: GCGCTACTTCCGAGACTACTT
	R: GGGCCTTATGCCAGGAAACT
qAgpat1	F: TAAGATGGCCTTCTACAACGGC
	R: CCATACAGGTATTTGACGTGGAG
qAgpat2	F: CAGCCAGGTTCTACGCCAAG
	R: TGATGCTCATGTTATCCACGGT
qPrdm16	F: CCACCAGCGAGGACTTCAC
	R: CCACCAGCGAGGACTTCAC
qPgc-1α	F: TATGGAGTGACATAGAGTGTGCT
	R: CCACTTCAATCCACCCAGAAAG
qFabp4	F: AAGGTGAAGAGCATCATAACCCT
	R: TCACGCCTTTCATAACACATTCC
qCox5b	F: TTCAAGGTTACTTCGCGGAGT
	R: CGGGACTAGATTAGGGTCTTCC
qCox7a	F: GCTCTGGTCCGGTCTTTTAGC
	R: GTACTGGGAGGTCATTGTCGG
qAtgl	F: CTGAGAATCACCATTCCCACATC
	R: CACAGCATGTAAGGGGGAGA
qHsl	F: TTCTCCAAAGCACCTAGCCAA
	R: TGTGGAAAACTAAGGGCTTGTTG
qLpl	F: GGGAGTTTGGCTCCAGAGTTT
	R: TGTGTCTTCAGGGGTCCTTAG
qBscl2	F: AATCATCTCCACTTCTTCACGCTC
	R: TCCAGTTGTTGGCACATACGAAT
**ChIP qPCR**	
Bscl2 TSS	F: AACAGGCAGCCGCCATCTT
	R: AGGCCCAGGGACAGCAGC
MG53	F: AGGGAGTGGGTAGGACAGCTAAATAT

	R: CAGGCTCAATGCAAGGGCAGGGA
